# Synthesis of Cobalt Bis(Dicarbollide)—Curcumin Conjugates for Potential Use in Boron Neutron Capture Therapy

**DOI:** 10.3390/molecules27144658

**Published:** 2022-07-21

**Authors:** Lyubov G. Dezhenkova, Anna A. Druzina, Yulia L. Volodina, Nadezhda V. Dudarova, Natalia A. Nekrasova, Olga B. Zhidkova, Mikhail A. Grin, Vladimir I. Bregadze

**Affiliations:** 1 Gause Institute of New Antibiotics, 11 B. Pirogovskaya Street, 119021 Moscow, Russia; dezhenkovalg@yahoo.com; 2 A.N. Nesmeyanov Institute of Organoelement Compounds, Russian Academy of Sciences, 28 Vavilov Str., 119991 Moscow, Russia; nadezjdino_96@mail.ru (N.V.D.); nekrasova_na@list.ru (N.A.N.); zolga57@mail.ru (O.B.Z.); bre@ineos.ac.ru (V.I.B.); 3 Blokhin Cancer Center, 24 Kashirskoye Shosse, 115478 Moscow, Russia; uvo2003@mail.ru; 4 M.V. Lomonosov Institute of Fine Chemical Technology, MIREA—Russian Technological University, 86 Vernadsky Av., 119571 Moscow, Russia; michael_grin@mail.ru

**Keywords:** curcumin, cobalt bis(dicarbollide), polyhedral boron compounds, cell viability, intracellular accumulation

## Abstract

A series of novel cobalt bis(dicarbollide)—curcumin conjugates were synthesized. Two conjugates were obtained through the nucleophilic ring-opening reaction of the 1,4-dioxane and tetrahydropyran derivatives of cobalt bis(dicarbollide) with the OH group of curcumin, and using two equiv. of the oxonium derivatives, two other conjugates containing two cobalt bis(dicarbollide) units per molecule were obtained. In contrast to curcumin, the conjugates obtained were found to be non-cytotoxic against both tumor and normal cell lines. The analysis of the intracellular accumulation of the conjugates by flow cytometry showed that all cobalt bis(dicarbollide)—curcumin conjugates entered HCT116 colorectal carcinoma cells in a time-dependent manner. New non-cytotoxic conjugates contain a large amount of boron atoms in the biomolecule and can potentially be used for further biological research into boron neutron capture therapy (BNCT).

## 1. Introduction

BNCT (boron neutron capture therapy) has recently been attracting attention as a non-invasive cancer therapy. The neutron capture reaction of ^10^B(n, α)^7^Li generates high linear energy transfer particles with a range of 5–9 µm, resulting in significant damage to boron-containing cells [1,2]. BNCT requires: (1) the low toxicity of boron-containing compounds; (2) their selective delivery in high concentration (>25 µg/g tissue) to the malignant tumor tissue, while the concentration of boron in the cells of the surrounding normal tissues should be maintained at a low level to minimize damage to normal tissues; and (3) the relatively rapid clearance of boron-containing compounds from blood and normal tissues with their persistence in the tumor for at least several hours during thermal neutron irradiation [3,4,5].

At the present time, the two boron compounds that have been extensively used in clinical BNCT trials are L-*p*-dihydroxy-borylphenylalanine (BPA) in the form of a fructose ester for increasing water solubility [6,7] and disodium mercaptoundecahydro-*closo*-dodecaborate (BSH) [8,9]. However, despite their clinical use, both BPA and BSH show low tumor-targeting selectivity and low retention time in tumors; therefore, many research groups have made great efforts to develop new, more effective BNCT-agents. Recently, α-D-mannopyranoside containing three *closo*-dodecaborate cages showed a broad intracellular distribution in vitro, a high uptake, a low toxicity in vivo and a good tumor-to-normal tissue accumulation ratio [10]. In an in vivo study, compared with clinically used fructose–BPA complexes, poly(vinyl alcohol)–BPA exhibited efficient tumor accumulation and prolonged tumor retention with quick clearance from the bloodstream and normal organs, and showed critically enhanced antitumor activity in BNCT [11].

One promising trend aimed at achieving the necessary therapeutic concentration of boron in the tumor is the use of polyhedral boron hydrides containing up to 18 boron atoms in the molecule [12]. Another promising trend is the development of various boronated biomolecules, such as amino acids [13], nucleosides [14], porphyries [15], and cholesterol [16], etc., which can be used as both boron host molecules and delivery agents. The high content of boron in the molecule, as well as its remarkable thermal and photochemical stabilities [17], have led to research into the use of cobalt bis(dicarbollide) anion and its derivatives as potential boron neutron capture therapy (BNCT) agents [18,19]. The constant interest in cobalt bis(dicarbollide) anion [3,3′-Co(1,2-C_2_B_9_H_11_)_2_]^–^ and its derivatives has resulted in some excellent contributions and important progress being made in the chemistry of polyhedral boron hydrides during the past few decades. Increasingly, research efforts have been dedicated to the application of [3,3′-Co(1,2-C_2_B_9_H_11_)_2_]^–^ in BNCT, as stable cobalt bis(dicarbollide) [17] shows resistance to catabolism and amphiphilicity [20,21], a good solubility of sodium salts, and low toxicity both in vitro [22,23] and in vivo [22,24]. It showed no signs of acute toxicity when intravenously [22] and intraperitoneally [24] injected into wild-type mice. It is known that bis(dicarbollide) cobalt can pass directly through synthetic lipid membranes [25,26,27] and accumulate in cells without disrupting membrane integrity [28].

Importantly, the choice of natural biomolecules linked to the polyhedral boron hydrides is demonstrated to be vitally important for the change in the antitumor activity of BNCT drugs based on the boron cluster. For several decades, herbal and natural compounds or their derivatives have attracted much attention as promising therapeutic agents for the treatment of human malignancies, especially cancers. Curcumin is a herbal supplement originating from turmeric (the root of the rhizome *Curcuma longa*) and belongs to the curcuminoids group, which are plant phenol metabolites [29,30]. Chemically, curcumin is a natural linear diarylheptanoid consisting of two aromatic rings which bind to a heptane with various substitutions. There is no doubt that curcumin plays a pivotal role in various biological processes because of its pharmaceutical benefits in remedying diseases. There is strong evidence that this polyphenol compound is capable of immense biological activity, including anti-oxidant, anti-inflammatory [31], cardioprotective [32], neuroprotective [33], as well as anti-cancer activity [34]. It is worth noting that the lipophilic properties of curcumin and its ability to cross the blood–brain barrier (BBB) make it an efficient therapeutic and protective agent in CNS-related disorders and malignancies [35,36,37]. At the time of our study, new research was published showing an innovative nanoparticle targeting folic acid containing both a Gd and a curcumin–boron complex, which proved to be an effective agent, opening up new perspectives for BNCT [38]. However, this curcumin–boron complex contains only one boron atom, and the derivatives of curcumin with polyhedral boron hydrides are not yet known. This motivated us to use cobalt bis(dicarbollide) containing about 1.5 times as much boron as BSH and 18 times more boron atoms than BPA. Thus, the aim of this study was to combine curcumin and non-cytotoxic cobalt bis(dicarbollide) containing a lot of boron atoms in the molecules that are necessary for BNCT. In this paper, we describe the synthesis of novel cobalt bis(dicarbollide)—curcumin conjugates with polyethylene glycol fragments in a spacer.

## 2. Results and Discussion

### 2.1. Synthesis of the Cobalt Bis(Dicarbollide)—Curcumin Conjugates ***4***–***7***

Recently, we designed a series of curcumin conjugated with cobalt bis(dicarbollide) and *closo*-dodecaborate using a “click” reaction [39]. It is known that polyethylene glycol fragments are widely used as a covalent modifier of biological macromolecules and particulates, as well as a linker for preparing bioconjugates with various biologically relevant molecules.

**Scheme 1:**
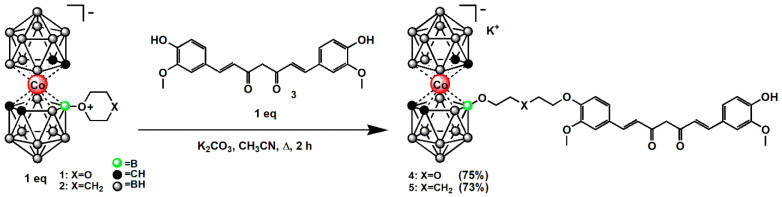
Synthesis of cobalt bis(dicarbollide)—curcumin conjugates with one metallacarborane moiety **4** and **5**.

One of the common methods used for the functionalization of polyhedral boron hydrides and their binding to different biomolecules is based on the nucleophilic ring opening of cyclic oxonium derivatives. The oxonium derivatives of cobalt bis(dicarbollide) react with alcohols to give corresponding substituted derivatives of cobalt bis(dicarbollide) [40]. In this study, we used cobalt bis(dicarbollide) derivatives with different oxonium cycles **1** and **2** and curcumin **3**. The conjugates of cobalt bis(dicarbollide) with curcumin were obtained in a simple, one-step procedure based on the nucleophilic ring-opening reactions of 1,4-dioxane and tetrahydropyran derivatives of cobalt bis(dicarbollide) **1** and **2** with the OH-group of curcumin **3**. It was found that compounds **1** and **2** reacted with curcumin **3** at a molar ratio of 1:1 in the presence of K_2_CO_3_ as a base in CH_3_CN under reflux to give the new corresponding boronated curcumin derivatives of the cobalt bis(dicarbollide). The reaction course of the preparation of all the desirable products was monitored by thin-layer chromatography using CH_2_Cl_2_-CH_3_CN (3:1) as an eluent. New monoanionic derivatives **4** and **5** were isolated as K-salts with good yields (73–75%) (Scheme 1).

**Scheme 2:**
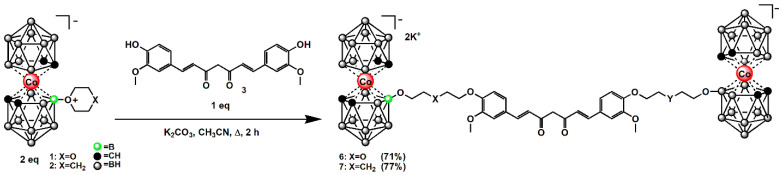
Synthesis of cobalt bis(dicarbollide)—curcumin conjugates with two metallacarborane moieties **6** and **7**.

The construction of boron-enriched systems has received considerable interest for use in BNCT, since one of the important requirements of BNCT is the synthesis of structures with a higher content of boron atoms in the molecule than in the clinically used compounds [5,6,41]. As mentioned above, functionalized cobalt bis(dicarbollide) can be used as a building block for the design and construction of such boron-containing compounds for various medical applications [42,43]. In this study, we combined two cobalt bis(dicarbollide) clusters into one molecule and obtained boron-enriched cluster compounds bearing a metallacarborane—curcumin conjugated system. As anticipated, an analogous nucleophilic ring opening of cyclic oxonium derivatives **1** and **2** with curcumin at a molar ratio of 2:1 generally led to the formation of dianionic products **6** and **7** as K-salts with good yields (71–77%) (Scheme 2).

The structures of the obtained conjugates **4–7** were confirmed by ^1^H-, ^11^B- and ^13^C-NMR; IR spectroscopy; and high-resolution mass spectrometry (see Supplementary Materials). The characteristic signals of the protons of the curcumin skeleton and the signals of boron atoms characteristic of the bis(dicarbollide) cage were found using ^1^H and ^11^B NMR spectroscopy.

It is also worth noting that the use of different cyclic esters of [3,3′-Co(C_2_B_9_H_11_)_2_]^–^ gave rise to spacers with a different degree of hydrophilicity or lipophilicity between the boron cage and the biological macromolecule. The ring opening of the 1,4-dioxane of [3,3′-Co(C_2_B_9_H_11_)_2_]^–^ with the OH-group of curcumin produced conjugates **4** and **6** with the hydrophilic spacers –(CH_2_CH_2_O)_2_–, whereas the tetrahydropyran oxonium rings opening produced conjugates **5** and **7** with lipophilic spacers –(CH_2_)_4-5_– between the boron cage and curcumin. These spacers can be considered long fragments, which have a high degree of flexibility and biocompatibility and can be introduced using a simple synthetic procedure.

### 2.2. Cell Viability Assay

We studied the influence of the novel cobalt bis(dicarbollide)—curcumin conjugates on the viability of two human tumor cell lines (HCT116 colorectal carcinoma and K562 chronic myelogenous leukemia) and non-malignant hFB-hTERT6 human skin fibroblasts. Doxorubicin (DOX) and curcumin were used as reference compounds. Curcumin was active against all tested cell lines, although its cytotoxicity for normal fibroblasts was slightly lower than for malignant cells. The cobalt bis(dicarbollide)—curcumin conjugates **4–7** turned out to be inactive. The results of this experiment are shown in Table 1.

### 2.3. Intracellular Drug Accumulation

**Figure 1:**
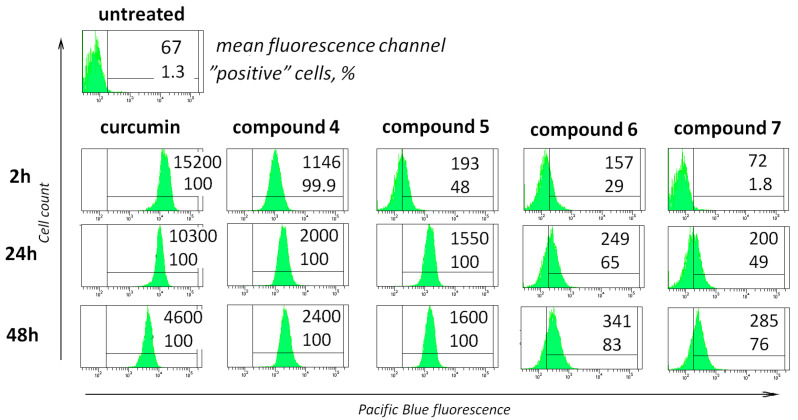
Intracellular accumulation of curcumin and cobalt bis(dicarbollide)—curcumin conjugates **4–7** in HCT116 colon carcinoma cells. Cells were treated with 25 μM of curcumin or conjugates of cobalt bis(dicarbollide) with curcumin **4–7** for the indicated time intervals, washed with saline and analyzed using flow cytometry. Percentage of “positive cells” and values of mean fluorescence channel were calculated from the respective histograms. One representative experiment out of three with similar results is shown.

To determine whether the lack of cytotoxicity is associated with poor cell penetration, we examined the time course of the intracellular drug accumulation using flow cytometry. This was possible due to the autofluorescence of tested compounds. We confirmed that all cobalt bis(dicarbollide)—curcumin conjugates **4–7** entered HCT116 cells in a time-dependent manner. Conjugate **4** penetrated cells better than the others (all the cells accumulated this compound after only 2 h of incubation); HCT116 incubation with compounds **6** and **7** for 48 h was not sufficient for complete accumulation (Figure 1). However, it can be argued that most of the cell population (80% and 75% for **6** and **7**, respectively) or even the entire population (for **4** and **5**) accumulate the compounds within 48 h. Curcumin demonstrated maximum cell-associated fluorescence after 2 h of incubation under the same conditions. Thus, compounds **4–7** penetrate cells without affecting their viability.

## 3. Materials and Methods

### 3.1. General Methods

1,4-Dioxane derivative of cobalt bis(dicarbollide) **1 [44]** and tetrahydropyran derivative of cobalt bis(dicarbollide) **2 [45]** were synthesized according to the published procedures. Curcumin (Acros Organics, Loughborough, U.K.) was used without further purification. CH_3_CN, CH_2_Cl_2_ and K_2_CO_3_ were commercially analytical-grade reagents. The reaction progress was monitored using thin-layer chromatography (Merck F245 silica gel on aluminum plates). Acros Organics silica gel (0.060–0.200 mm) was used for column chromatography. The NMR spectra at 400.1 MHz (^1^H), 128.4 MHz (^11^B) and 100.0 MHz (^13^C) were recorded with a Varian Inova 400 and a Bruker Avance-400 spectrometer. The residual signal of the NMR solvent relative to Me_4_Si was taken as the internal reference for ^1^H- and ^13^C-NMR spectra. ^11^B-NMR spectra were referenced using BF_3_ × Et_2_O as an external standard. Infrared spectra were recorded on a Spectra SF 2000 instrument. High-resolution mass spectra (HRMS) were measured on a mictOTOF II instrument using electrospray ionization (ESI). The measurements were carried out in negative ion mode (interface capillary voltage 3200 V; mass range from *m/z* 50 to *m/z* 3000).

General Procedure for the Synthesis of the Cobalt Bis(Dicarbollide)—Curcumin Conjugates **4–7:**

The corresponding oxonium derivative of cobalt bis(dicarbollide) **1** or **2** (1.0 or 2.0 equiv.) and K_2_CO_3_ (5 equiv.) were added to a solution of curcumin **3** (1.0 equiv.) in CH_3_CN (30 mL). The reaction mixture was heated under reflux conditions for 2 h. After cooling to room temperature, the precipitate was filtered off and the solvent was evaporated in vacuo. The residue was purified by column chromatography on silica with a mixture of dichloromethane and acetonitrile (3/1 *v*/*v*) as an eluent. The major fraction was collected and vacuum-dried to give the target products **4–7** as an orange foam.

### 3.2. Synthesis of (8-[(H(CH_2_[COCH=CH(OCH_3_)C_6_H_3_O]_2_))-((CH_2_)_2_O)_2_]-3,3′-Co(1,2-C_2_B_9_H_10_)(1′,2′-C_2_B_9_H_11_))K (***4***)

Compound **4** was prepared according to the general procedure using the 1,4-dioxane derivative of cobalt bis(dicarbollide) **1** (0.20 g, 0.48 mmol), curcumin **3** (0.18 g, 0.48 mmol) and K_2_CO_3_ (0.34 g, 2.44 mmol). Yield: 0.30 g (75%). ^1^H NMR (400 MHz, CD_3_CN-*d*_3_): δ 7.59 (2H, d, 2×-C*H*=CH-, *J* = 18.0 Hz), 7.26 (2H, d, 2×-C*H*=C *in phehyl, J* = 8.2 Hz), 7.21 (1H, d, -C*H*=C *in phehyl*, *J* = 3.7 Hz, 7.14 (1H, d, -C*H*=C *in phehyl*, *J* = 8.2 Hz), 6.99, (1H, m, -CH=C*H*-), 6.86 (1H, d, -C*H*=CH-, *J* = 8.6 Hz), 6.71 (2H, m, 2×-C*H*=C *in phehyl*), 5.94 (1H, s, C*H*), 4.17 (2H, s, 2×C*H*_carb_), 4.12 (2H, s, 2×C*H*_carb_), 4.03 (2H, s, -C*H_2_*O), 3.91 (6H, s, 2× C*H_3_*OC_6_H_3_-), 3.82 (2H, s, -C*H_2_*O), 3.70 (2H, s, -C*H_2_*O), 3.60 (2H, s, -C*H_2_*O), 1.5–0.5 (br.m, BH) ppm; ^11^B NMR (128 MHz, CD_3_CN-*d*_3_) δ 24.1 (1B, s), 5.6 (1B, d, *J* = 130 Hz), 0.0 (1B, d, *J* = 143 Hz), −2.7 (1B, d, *J* = 129 Hz), −5.2 (2B, d, *J* = 177 Hz), −7.3 (6B, d, *J* = 135 Hz), −17.4 (2B, d, *J* = 131 Hz), −20.7 (2B, d, *J* = 138 Hz), −22.4 (1B, d, *J* unsolved), −28.2 (1B, d, *J* unsolved) ppm. ^13^C NMR (101 MHz, CD_3_CN-*d*_3_): 184.0 (-C=O), 183.2 (-C=O), 149.6 (=*C*-O-CH_3_ *in phehyl*), 149.0 (=*C*-O-CH_3_ *in phehyl*), 148.6 (=*C*-O-CH_2_- *in phehyl*), 147.6 (=*C*-OH *in phehyl*), 140.5 (-*C*H=CH-), 139.9 (-*C*H=CH-), 128.6 (=*C*H-C=O), 127.5 (=*C*H-C=O), 123.1 (=*C*-CH- *in phehyl*), 122.8 (-*C*=CH- *in phehyl*), 122.4 (=*C*H-CH- *in phehyl*), 121.7 (-*C*H=CH- *in phehyl*), 115.0 (=*C*H-C-O-CH_3_), 112.6 (-*C*H=C-O-CH_3_), 110.5 (-*C*H=C-OH), 110.4 (=*C*H-C-OH), 101.3 (*C*H_2_-C=O), 71.5 (O*C*H_2_), 68.6 (O*C*H_2_), 68.4 (O*C*H_2_), 67.6 (O*C*H_2_), 55.8 (O-CH_3_), 55.7 (O-CH_3_), 52.7 (2×*C*H_carb_), 46.8 (2×*C*H_carb_). IR (solid): ν˜ = 2662 cm^−1^ (BH), 1534 cm^−1^ (C=O). HRMS (ESI) m/z for [C_29_H_48_B_18_CoO_8_]^−^ calcd 778.4471, found: 778.4490.

### 3.3. Synthesis of (8-[(H(CH_2_[COCH=CH(OCH_3_)C_6_H_3_O]_2_))-(CH_2_)_5_O]-3,3′-Co(1,2-C_2_B_9_H_10_)(1′,2′-C_2_B_9_H_11_))K (***5***)

Compound **5** was prepared according to the general procedure using the tetrahydropyran derivative of cobalt bis(dicarbollide) **2** (0.20 g, 0.48 mmol), curcumin **3** (0.18 g, 0.48 mmol) and K_2_CO_3_ (0.34 g, 2.44 mmol). Yield: 0.29 g (73%). ^1^H NMR (400 MHz, CD_3_CN-*d*_3_): δ 7.58 (2H, d, 2×-C*H*=CH-, *J* = 15.9 Hz), 7.25 (2H, d, 2×-C*H*=C *in phehyl, J* = 8.5 Hz), 7.16 (1H, d, -C*H*=C *in phehyl*, *J* = 3.7 Hz, 7. 03 (1H, d, -C*H*=C *in phehyl*, *J* = 8.2 Hz), 6.94, (1H, m, -CH=C*H*-), 6.86 (1H, d, -C*H*=CH-, *J* = 8.6 Hz), 6.69 (2H, m, 2×-C*H*=C *in phehyl*), 5.92 (1H, s, C*H*), 4.22 (2H, s, 2×C*H*_carb_), 4.09 (2H, s, 2×C*H*_carb_), 4.00 (2H, s, -C*H_2_*O), 3.91 (6H, s, 2× C*H_3_*OC_6_H_3_-), 3.87 (2H, s, -C*H_2_*O), 3.48 (2H, s, -C*H_2_*O), 1.75 (2H, s, -C*H_2_*O), 1.50 (2H, s, -C*H_2_*O), 1.5–0.5 (br.m, BH) ppm; ^11^B NMR (128 MHz, CD_3_CN-*d*_3_) δ 23.7 (1B, s), 4.5 (1B, d, *J* = 134 Hz), 0.1 (1B, d, *J* = 142 Hz), −2.6 (1B, d, *J* = 150 Hz), −5.0 (2B, d, *J* = 132 Hz), −7.7 (6B, d, *J* = 123 Hz), −17.5 (2B, d, *J* = 154 Hz), −20.4 (2B, d, *J* = 155 Hz), −22.1 (1B, d, *J* unsolved), −28.6 (1B, d, *J* unsolved) ppm. ^13^C NMR (101 MHz, CD_3_CN-*d*_3_): 183.7 (-C=O), 183.6 (-C=O), 150.9 (=*C*-O-CH_3_ *in phehyl*), 149.5 (=*C*-O-CH_3_ *in phehyl*), 148.6 (=*C*-O-CH_2_- *in phehyl*), 147.6 (=*C*-OH *in phehyl*), 140.4 (-*C*H=CH-), 140.3 (-*C*H=CH-), 129.9 (=*C*H-C=O), 127.8 (=*C*H-C=O), 127.5 (=*C*-CH- *in phehyl*), 123.1 (-*C*=CH- *in phehyl*), 122.9 (=*C*H-CH- *in phehyl*), 121.9 (-*C*H=CH- *in phehyl*), 121.7 (=*C*H-C-O-CH_3_), 115.0 (-*C*H=C-O-CH_3_), 112.6 (-*C*H=C-OH), 110.5 (=*C*H-C-OH), 101.2 (*C*H_2_-C=O), 68.8 (2×O*C*H_2_), 55.8 (O-CH_3_), 55.6 (O-CH_3_), 53.6 (2×*C*H_carb_), 46.6 (2×*C*H_carb_), 31.5 (*C*H_2_), 28.8 (*C*H_2_), 22.4 (*C*H_2_). IR (solid): ν˜ = 2543 cm^−1^ (BH), 1483 cm^−1^ (C=O). HRMS (ESI) m/z for [C_30_H_50_B_18_CoO_7_]^-^ calcd 776.4678, found: 776.4695.

### 3.4. Synthesis of (8-[(H(CH_2_[COCH=CH(OCH_3_)C_6_H_3_O]_2_))-((CH_2_)_2_O)_2_]-{3,3′-Co(1,2-C_2_B_9_H_10_)(1′,2′-C_2_B_9_H_11_)}_2_)K_2_ (***6***)

Compound **6** was prepared according to the general procedure using the 1,4-dioxane derivative of cobalt bis(dicarbollide) **1** (0.20 g, 0.48 mmol), curcumin **3** (0.09 g, 0.24 mmol) and K_2_CO_3_ (0.17 g, 1.22 mmol). Yield: 0.22 g (71%). ^1^H NMR (400 MHz, acetone-*d*_6_): δ 7.64 (2H, d, 2×-C*H*=CH-, *J* = 15.08 Hz), 7.37 (2H, s, 2×-C*H*=C *in phehyl*), 7.27 (2H, d, -C*H*=C *in phehyl*, *J* = 9.9 Hz), 7.09 (2H, d, 2×-C*H*=CH *in phehyl*, *J* = 8.4 Hz), 6.79, (1H, d, -CH=C*H*-, *J* = 15.08 Hz), 6.04 (1H, s, C*H*), 4.25 (12H, m, 8×C*H*_carb_, 2×-C*H_2_*O), 3.92 (10H, m, 2× C*H_3_*OC_6_H_3_, 2×-C*H_2_*O), 3.67 (8H, m, 4×-C*H_2_*O), 1.5–0.5 (br.m, BH) ppm; ^11^B NMR (128 MHz, acetone-*d*_6_) δ 23.3 (2B, s), 4.5 (2B, d, *J* = 140 Hz), 0.4 (2B, d, *J* = 167 Hz), −2.6 (2B, d, *J* = 131 Hz), −4.6 (4B, d, *J* = 126 Hz), −7.2 (6B, d, *J* = 125 Hz), −7.9 (6B, d, *J* = 132 Hz), −17.1 (4B, d, *J* = 169 Hz), −20.4 (4B, d, *J* = 163 Hz), −22.0 (2B, d, *J* unsolved), −28.2 (2B, d, *J* = 141 Hz) ppm. ^13^C NMR (101 MHz, acetone-*d*_6_): 183.6 (2×-C=O), 150.4 (2×=*C*-O-CH_3_ *in phehyl*), 149.6 (2×=*C*-O-CH_2_- *in phehyl*), 140.2 (2×=*C*-OH *in phehyl*), 128.5 (2×-*C*H=CH-), 122.7 (2×=*C*H-C=O), 122.3 (2×=*C*-CH- *in phehyl*), 113.0 (2×=*C*H-CH- *in phehyl*), 110.7 (2×=*C*H-C-O-CH_3_), 101.0 (2×-*C*H=C-OH), 71.9 (*C*H_2_-C=O), 69.0 (2×O*C*H_2_), 68.4 (2×O*C*H_2_), 68.1 (2×O-*C*H_3_), 55.5 (2×OCH_2_), 53.8 (4×*C*H_carb_), 46.5 (4×*C*H_carb_), 33.6 (2×O*C*H_2_). IR (solid): ν˜ = 2545 cm^−1^ (BH), 1496 cm^−1^ (C=O). HRMS (ESI) m/z for [C_37_H_76_B_36_Co_2_O_10_]^2-^ calcd 593.8859, found: 593.8872.

### 3.5. Synthesis of (8-[(H(CH_2_[COCH=CH(OCH_3_)C_6_H_3_O]_2_))-(CH_2_)_5_O]-{3,3′-Co(1,2-C_2_B_9_H_10_)(1′,2′-C_2_B_9_H_11_)}_2_)K_2_ (***7***)

Compound **7** was prepared according to the general procedure using the tetrahydropyran derivative of cobalt bis(dicarbollide) **2** (0.17 g, 0.42 mmol), curcumin **3** (0.08 g, 0.21 mmol) and K_2_CO_3_ (0.15 g, 1.10 mmol). Yield: 0.20 g (77 %). ^1^H NMR (400 MHz, acetone-*d*_6_): δ 7.73 (2H, d, 2×-C*H*=CH-, *J* = 15.8 Hz), 7.38 (2H, s, 2×-C*H*=C *in phehyl*), 7.33 (2H, d, -C*H*=C *in phehyl*, *J* = 8.3 Hz), 7.09 (2H, d, 2-C*H*=CH *in phehyl*, *J* = 8.3 Hz), 6.85, (1H, d, -CH=C*H*-, *J* = 15.8 Hz), 6.08 (1H, s, C*H*), 4.36 (4H, s, 4×C*H*_carb_,), 4.23 (4H, s, 4×C*H*_carb_), 4.15 (4H, t, 2×-C*H_2_*O, *J* = 6.7 Hz), 4.00 (6H, s, 2×C*H_3_*OC_6_H_3_), 3.61 (4H, t, 2×-C*H_2_*O, *J* = 5.9 Hz), 1.90 (4H, m, 2×-C*H_2_*), 1.62 (8H, m, 4×-C*H_2_*), 1.5–0.5 (br.m, BH) ppm; ^11^B NMR (128 MHz, acetone-*d*_6_) δ 23.8 (2B, s), 4.4 (2B, d, *J* = 112 Hz), 0.0 (2B, d, *J* = 146 Hz), −2.8 (2B, d, *J* = 131 Hz), −5.0 (4B, d, *J* = 160 Hz), −7.9 (6B, d, *J* = 107 Hz), −8.8 (6B, d, *J* = 114 Hz), −17.4 (4B, d, *J* = 169 Hz), −20.4 (4B, d, *J* = 170 Hz), −22.3 (2B, d, *J* unsolved), −29.0 (2B, d, *J* unsolved) ppm. ^13^C NMR (101 MHz, CD_3_CN-*d*_3_): 183.8 (2×-C=O), 151.0 (2×=*C*-O-CH_3_ *in phehyl*), 149.7 (2×=*C*-O-CH_2_- *in phehyl*), 140.4 (2×=*C*-OH *in phehyl*), 128.0 (2×-*C*H=CH-), 123.0 (2×=*C*H-C=O), 122.0 (2×=*C*-CH- *in phehyl*), 112.7 (2×=*C*H-CH- *in phehyl*), 110.6 (2×=*C*H-C-O-CH_3_), 101.4 (2×-*C*H=C-OH), 68.9 (*C*H_2_-C=O), 55.6 (2×O*C*H_2_), 53.7 (4×*C*H_carb_), 46.7 (4×*C*H_carb_), 31.6 (2×O*C*H_2_), 28.9 (2×*C*H_2_), 46.5 (2×*C*H_2_), 22.5 (2×*C*H_2_). IR (solid): ν˜ = 2549 cm^−1^ (BH), 1523 cm^−1^ (C=O). HRMS (ESI) m/z for [C_39_H_80_B_36_Co_2_O_8_]^2−^ calcd 591.9080, found: 591.9028.

### 3.6. Biological Testing

#### 3.6.1. Cell Cultures and Viability Assay

Cell lines HCT116 human colorectal carcinoma and K562 human chronic myelogenous leukemia were from American Type Culture Collection, USA. Cell line hFB-hTERT6 (non-malignant human skin fibroblasts) was obtained via a lentiviral transduction of full-length TERT gene under a cytomegalovirus promoter (generated at Engelhardt Institute of Molecular Biology, Moscow by Dr. E. Dashinimaev; gift of Prof. A. Shtil).

Adherent cells (HCT116 and hFB-hTERT6) were cultured in Dulbecco’s modified Eagle’s medium (PanEco, Russia, Moscow) supplemented with 10% fetal calf serum (HyClone-Cytiva, Marlborough, MA, USA), 2 mM L-glutamine, 100 U/mL penicillin, and 100 mg/mL streptomycin at 37 °C, 5% CO_2_ in a humidified atmosphere. Suspension cells (K562) were propagated in RPMI-1640 (PanEco, Russia, Moscow) with the same supplements. Cells in logarithmic phase were used in all experiments. All compounds were dissolved in DMSO as 10 mM stock solutions followed by serial dilutions in culture medium immediately before experiments.

The cytotoxicity was determined in a formazan assay (MTT-test) [46] using the standard method. Briefly, cells (5 × 10^3^ in 190 µL of culture medium) were plated into a 96-well plate (NUNC, Roskilde, Denmark) and treated with 0.1% DMSO (vehicle control) or with tested compounds (0.10–50 µM; each concentration in duplicate) and held for 72 h at 37 °C, 5% CO_2_ in a humidified atmosphere. Cells without drug served as a control. On completion of the drug exposure, 20 µL of aqueous MTT solution (Sigma, Burlington, MA, USA; 5 mg/mL) was added to each well for an additional 2–3 h. Then, media were removed from the cells (suspension cells K562 were pre-centrifuged on a plate centrifuge (Thermo FS, Waltham, MA, USA)), 150 µL DMSO was added for formazan dissolution, and the absorbance at 570 nm was measured using the microplate spectrophotometer ELx800 (BioTek, Santa Clara, CA, USA). The cytotoxic efficiency was calculated as the ratio of absorbance values in wells with drug-treated cells to those in wells with untreated control cells. The absorbance value in the control wells was taken as 100%. Dose–response curves were plotted and analyzed. The IC_50_ (50% growth inhibitory concentration) was defined as the concentration of the compound that inhibited MTT conversion by 50%.

#### 3.6.2. Intracellular Drug Accumulation

The HCT116 cells (2 × 10^5^ in 2 mL of medium) were treated with 25 µM of curcumin or cobalt bis(dicarbollide)—curcumin conjugates **4**–**7** for 2, 24 and 48 h; trypsinized; resuspended in PBS on ice; and immediately analyzed using flow cytometry on a BD FACSCanto II (BD Biosciences, Franklin Lakes, NJ, USA) in the Pacific Blue channel (filter 450/50). The cell-associated fluorescence of tested compounds was measured as the percentage of cells with fluorescence above the basal level (“positive cells”) and as values of the mean fluorescence channel (MFC). A total of 10,000 ‘events’ were acquired for each sample. Data were analyzed using the FACSDiva program (BD Biosciences).

## 4. Conclusions

The nucleophilic ring-opening reaction of 1,4-dioxane and tetrahydropyran derivatives of cobalt bis(dicarbollide) with curcumin in acetonitrile in the presence of K_2_CO_3_ gives the corresponding products containing one or two boron clusters in molecules depending on the initial ratio of reagents. The novel compounds did not demonstrate antiproliferative activity against two tumor and one non-tumor cell lines of different tissue origin. The time course of the intracellular accumulation of the drugs was studied using flow cytometry. It was shown that all cobalt bis(dicarbollide)—curcumin conjugates entered HCT116 cells in a time-dependent manner. Cobalt bis(dicarbollide)—curcumin conjugate with one metallacarborane moiety prepared from 1,4-dioxane derivative penetrates cells better than others. Thus, these novel non-cytotoxic cobalt bis(dicarbollide)—curcumin conjugates containing a large amount of boron atoms in the biomolecule can potentially be used for further biological research into BNCT.

## Figures and Tables

**Table 1:** IC_50_ values for cells treated with novel cobalt-curcumin conjugates **4–7** (MTT-test results, 72 h exposure).

Cell Line	IC_50_, µM					
	Curcumin	4	5	6	7	Dox (Control)
K562	17.0 ± 2.0 ^a^	>50	>50	>50	>50	0.20 ± 0.02
Fibroblasts	23.0 ± 2.5	>50	>50	>50	>50	0.30 ± 0.04
HCT116	11.5 ± 1.4	>50	>50	>50	>50	0.40 ± 0.05

^a^ Mean ± S.D. of 3 independent experiments.

## Data Availability

The data presented in this study are available in the Supplementary Materials.

## Supplementary Materials

The following supporting information can be downloaded at: https://www.mdpi.com/article/10.3390/molecules27144658/s1. Figures S1–S4: ESI-HRMS spectra of compounds **4–7**; Figures S5–S8: IR spectra of compounds **4–7**; Figures S9–S24: ^1^H, ^11^B{^1^H}, ^11^B and ^13^C spectra of compounds **4–7**.
