# Supplementary material for: Synthesis of Cobalt Bis(Dicarbollide)—Curcumin Conjugates for Potential Use in Boron Neutron Capture Therapy
Source: Molecules. 2022 Jul 21;27(14):4658. doi: 10.3390/molecules27144658 (PMC9324984; doi:10.3390/molecules27144658)
Supplement: Supplementary file 1 [file molecules-27-04658-s001.zip › molecules-1827329-supplementary.pdf]

SUPPORTING INFORMATION

Synthesis of Cobalt Bis(Dicarbollide)—Curcumin Conjugates for Potential Use in Boron Neutron Capture Therapy

Lyubov G. Dezhenkova 1, Anna A. Druzina 2,* , Yulia L. Volodina 3, Nadezhda V. Dudarova 2 , Natalia A. Nekrasova 2,4, Olga B. Zhidkova 2, Mikhail A. Grin 4 and Vladimir I. Bregadze 2

1 Gause Institute of New Antibiotics, 11 B. Pirogovskaya Street, 119021 Moscow, Russia; dezhenkovalg@yahoo.com

2 A.N. Nesmeyanov Institute of Organoelement Compounds, Russian Academy of Sciences, 28 Vavilov Str.,

119991 Moscow, Russia; nadezjdino_96@mail.ru (N.V.D.); nekrasova_na@list.ru (N.A.N.);

zolga57@mail.ru (O.B.Z.); bre@ineos.ac.ru (V.I.B.)

3 Blokhin Cancer Center, 24 Kashirskoye Shosse, 115478 Moscow, Russia; uvo2003@mail.ru

4 M.V. Lomonosov Institute of Fine Chemical Technology, MIREA—Russian Technological University, 86 Vernadsky Av., 119571 Moscow, Russia; michael_grin@mail.ru

* Correspondence: ilinova_anna@mail.ru; Tel.: +7-926-404-5566

^{1}H, ^{11}B and ^{13}C NMR, IR and high-resolution mass spectra of compounds **4-7**

Display Report

Analysis Info

Analysis Name	D:\Data\Chizhov\INEOS\Druzina\da-043_&clb-.d	Acquisition Date	24.05.2022 16:09:46
Method	tune_wide_neg.m	Operator	BDAL@DE
Sample Name	/CHIZ DA-043	Instrument / Ser#	maXis 43
Comment	CH3OH 100 %, dil. 200, calibrant added		

Acquisition Parameter

Source Type	ESI	Ion Polarity	Negative	Set Nebulizer	0.4 Bar
Focus	Active			Set Dry Heater	180 °C
Scan Begin	50 m/z	Set Capillary	3000 V	Set Dry Gas	4.0 l/min
Scan End	3000 m/z	Set End Plate Offset	-500 V	Set Divert Valve	Waste

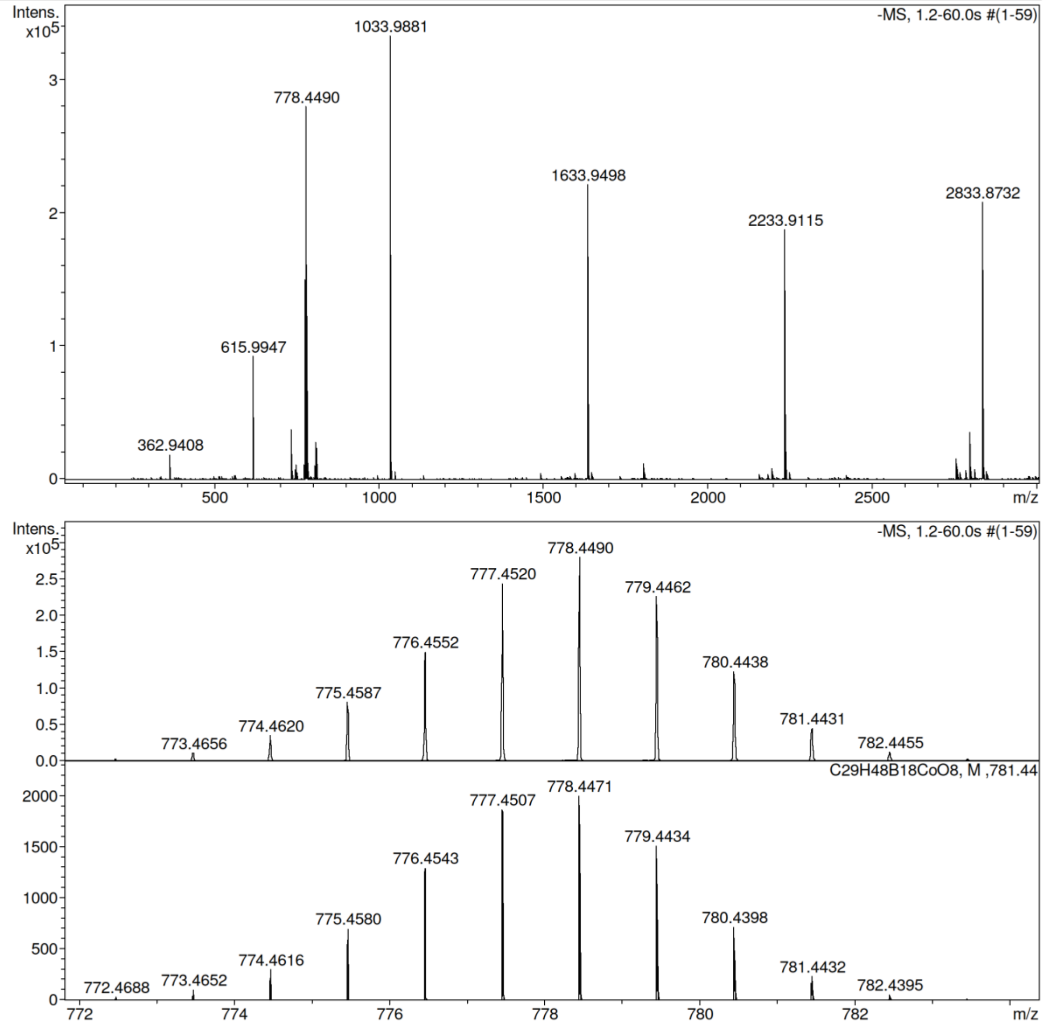

Bruker Compass DataAnalysis 4.0 printed: 24.05.2022 16:31:14 Page 1 of 1

Figure S1. ESI-HRMS spectrum of compound **4**

Display Report

Analysis Info

Analysis Name	D:\Data\Chizhov\INEOS\Druzina\da-042_&clblow-.d	Acquisition Date	24.05.2022 16:01:27
Method	tune_low_neg_1050.m	Operator	BDAL@DE
Sample Name	/CHIZ DA-042	Instrument / Ser#	maXis 43
Comment	CH3OH 100 %, dil. 200, calibrant added		

Acquisition Parameter

Source Type	ESI	Ion Polarity	Negative	Set Nebulizer	0.4 Bar
Focus	Not active			Set Dry Heater	180 °C
Scan Begin	50 m/z	Set Capillary	2000 V	Set Dry Gas	4.0 l/min
Scan End	1050 m/z	Set End Plate Offset	-500 V	Set Divert Valve	Source

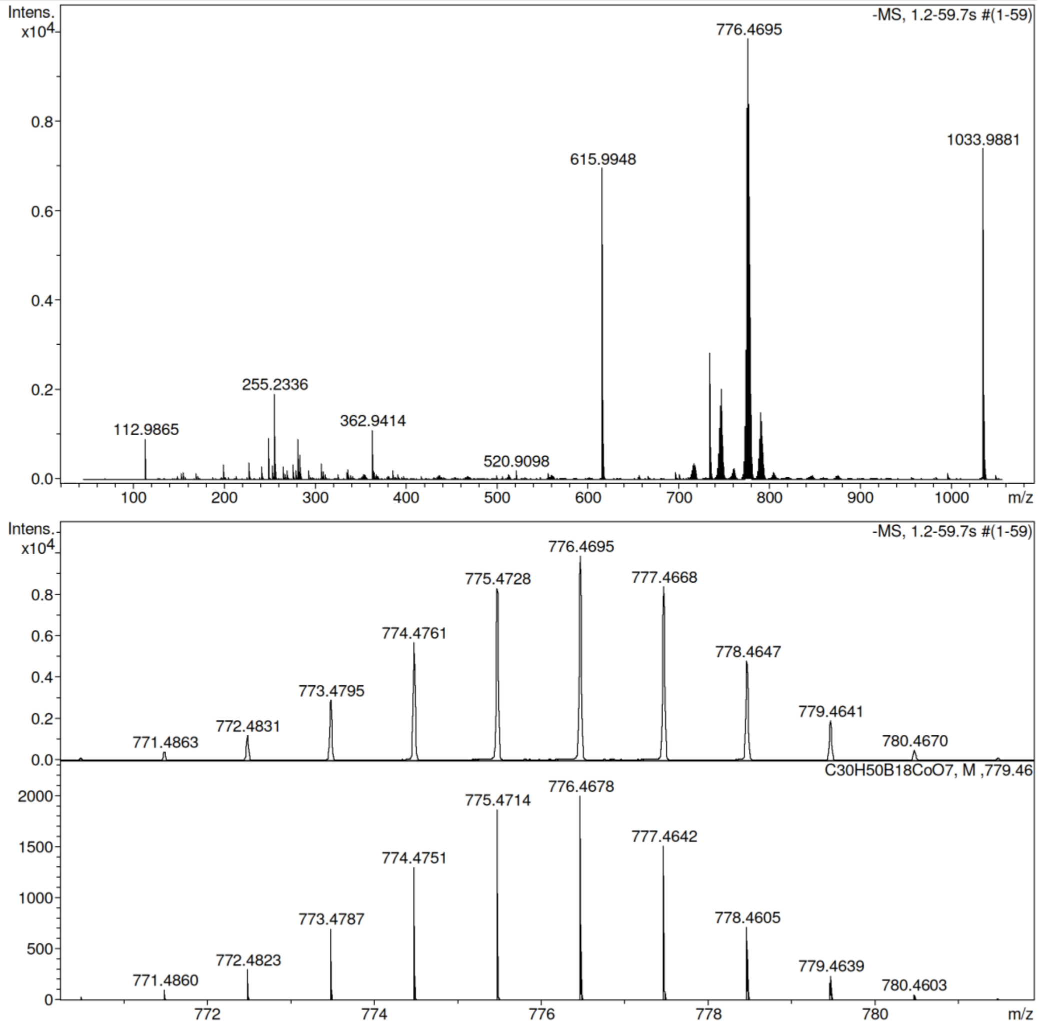

Bruker Compass DataAnalysis 4.0 printed: 24.05.2022 16:27:18 Page 1 of 1

Figure S2. ESI-HRMS spectrum of compound **5**

Display Report

Analysis Info

Analysis Name	D:\Data\Chizhov\INEOS\Druzina\da-044_&clb-.d	Acquisition Date	24.05.2022 16:17:44
Method	tune_wide_neg.m	Operator	BDAL@DE
Sample Name	/CHIZ DA-044	Instrument / Ser#	maXis 43
Comment	CH3OH 100 %, dil. 200, calibrant added		

Acquisition Parameter

Source Type	ESI	Ion Polarity	Negative	Set Nebulizer	0.4 Bar
Focus	Active			Set Dry Heater	180 °C
Scan Begin	50 m/z	Set Capillary	3000 V	Set Dry Gas	4.0 l/min
Scan End	3000 m/z	Set End Plate Offset	-500 V	Set Divert Valve	Waste

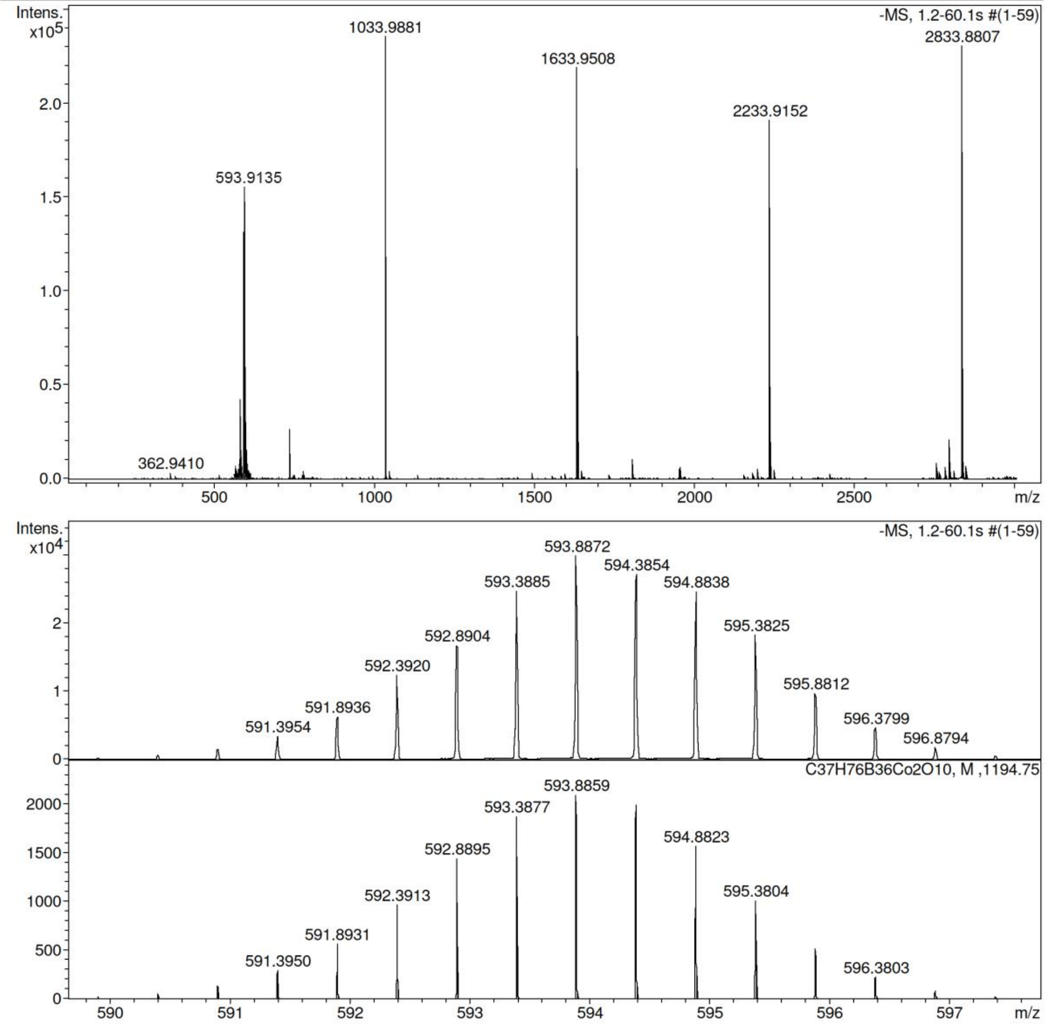

Figure S3. ESI-HRMS spectrum of compound **6**

Display Report

Analysis Info

Analysis Name	D:\Data\Chizhov\INEOS\Bregadze\Druzina\Jan_17_2022\da-045-2_&clb-.d	Acquisition Date	17.01.2022 19:26:09
Method	tune_wide_neg.m	Operator	BDAL@DE
Sample Name	/CHIZ DA-045-2	Instrument / Ser#	micrOTOF 10248
Comment	CH3CN 100 %, dil. 20, calibrant added		

Acquisition Parameter

Source Type	ESI	Ion Polarity	Negative	Set Nebulizer	0.4 Bar
Focus	Not active			Set Dry Heater	180 °C
Scan Begin	50 m/z	Set Capillary	3200 V	Set Dry Gas	4.0 l/min
Scan End	3000 m/z	Set End Plate Offset	-500 V	Set Divert Valve	Waste

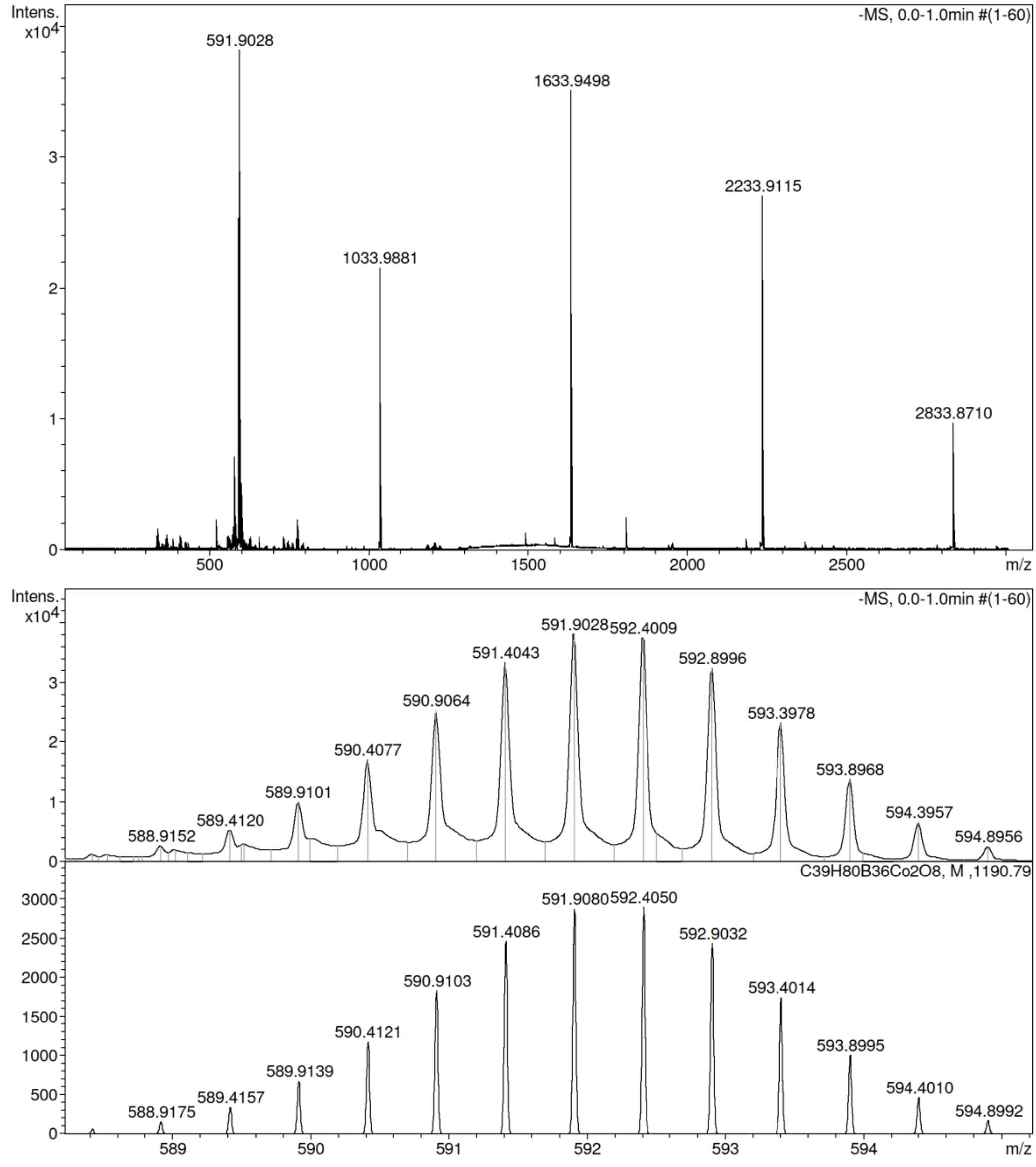

Bruker Compass DataAnalysis 4.0 printed: 17.01.2022 19:41:02 Page 1 of 1

Figure S4. ESI-HRMS spectrum of compound **7**

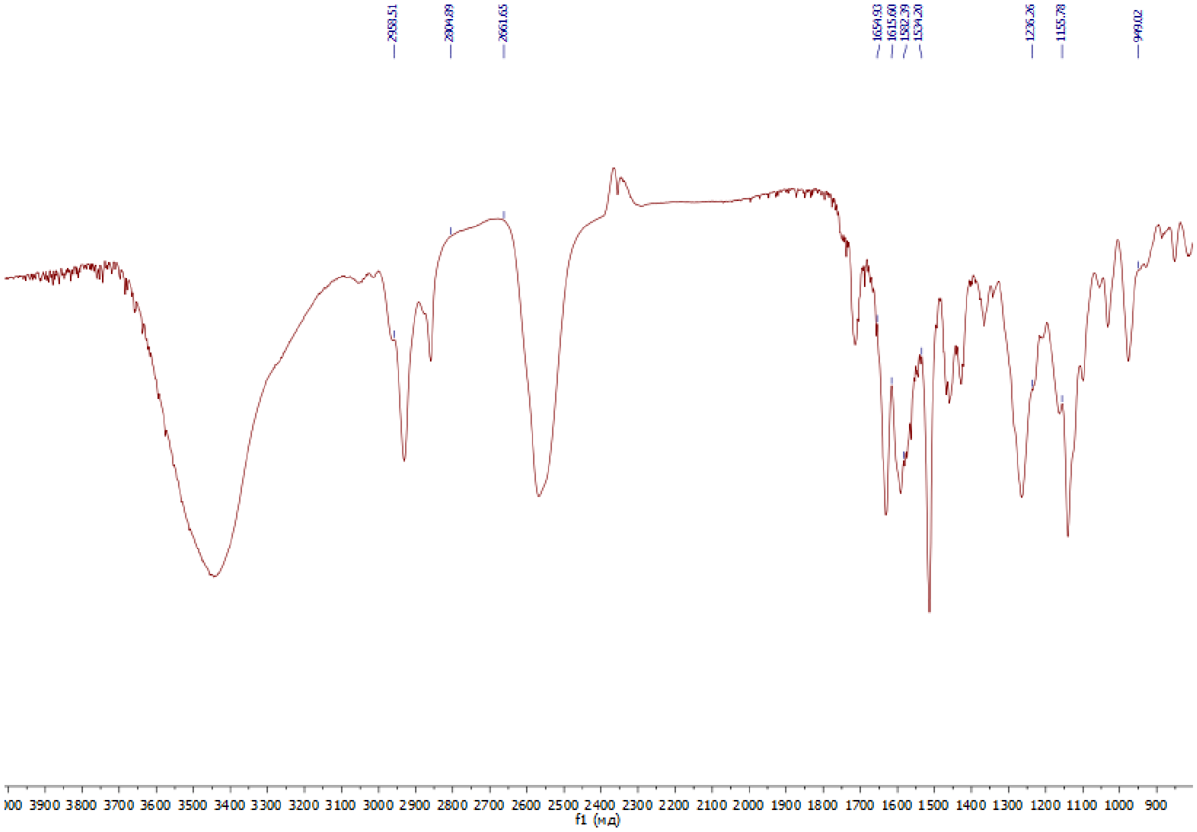

Figure S5. IR spectrum of compound **4**

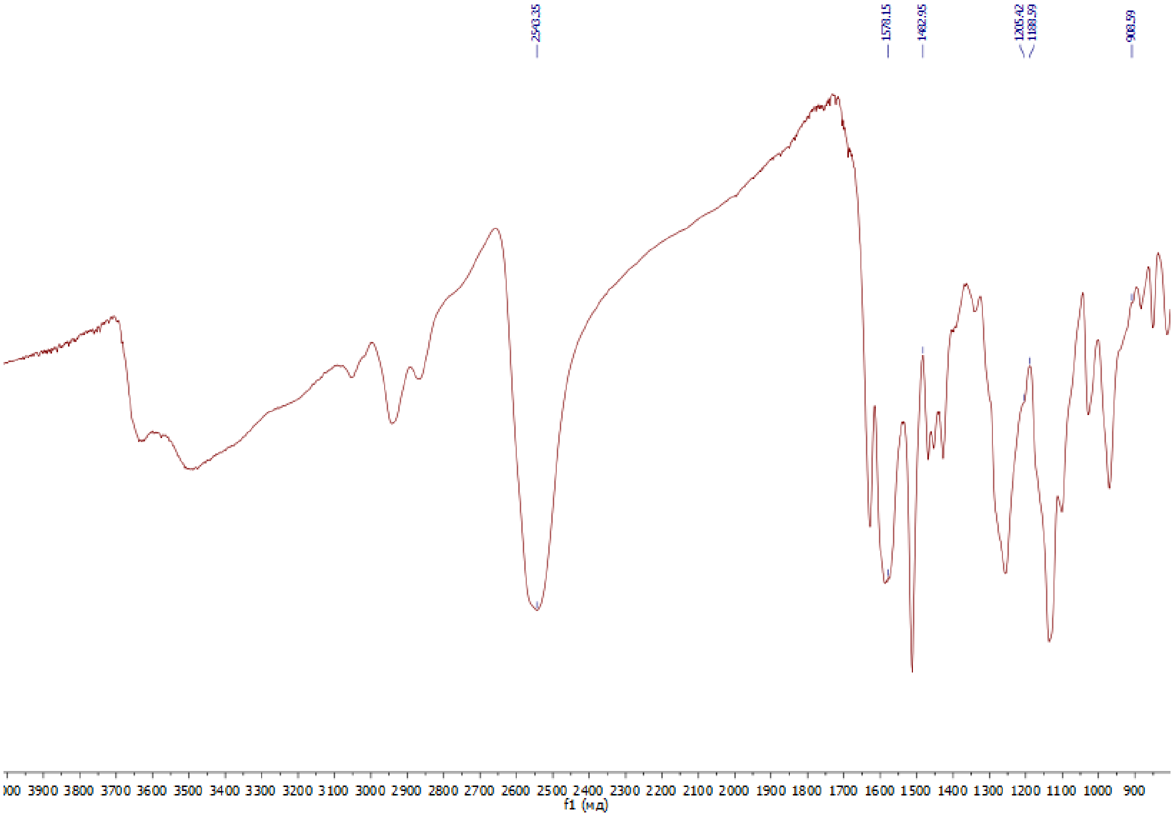

Figure S6. IR spectrum of compound **5**

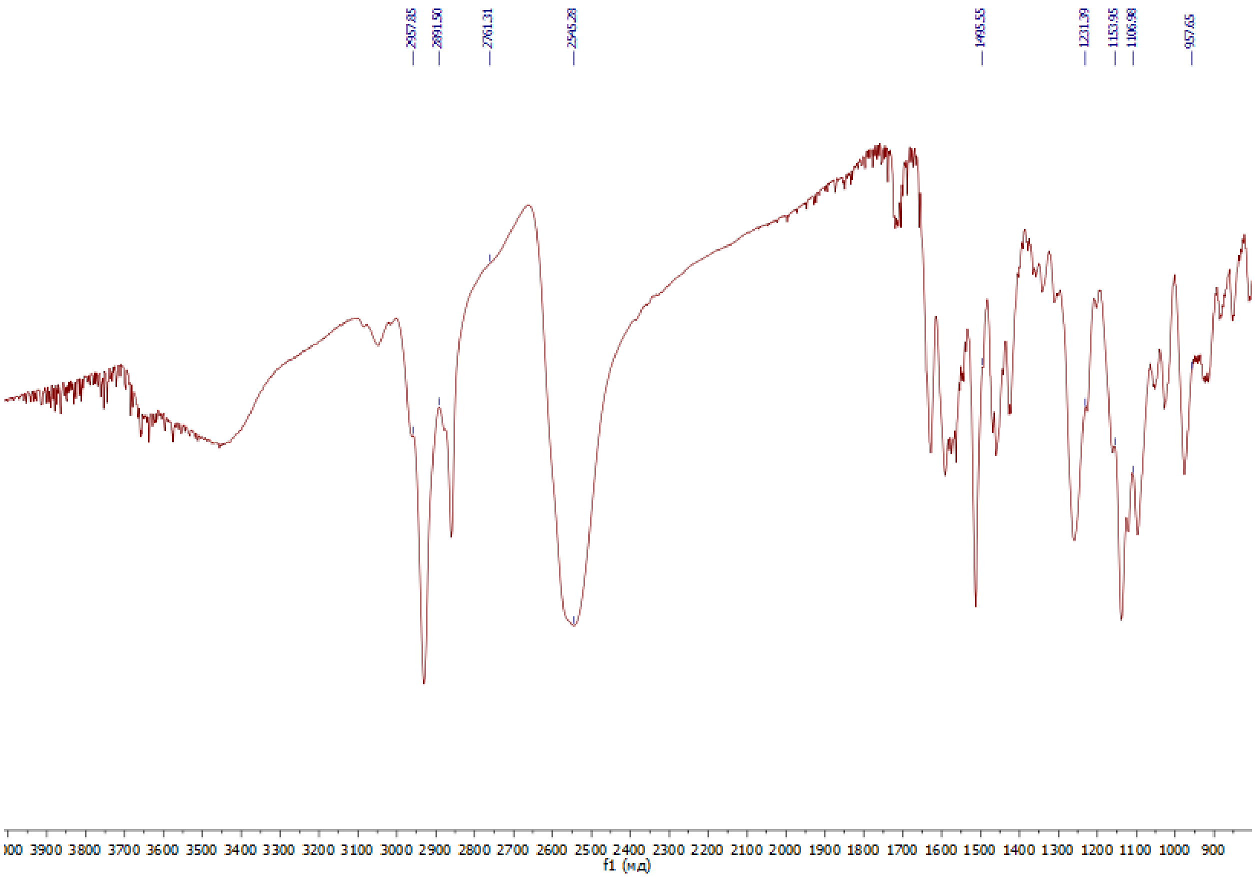

Figure S7. IR spectrum of compound **6**

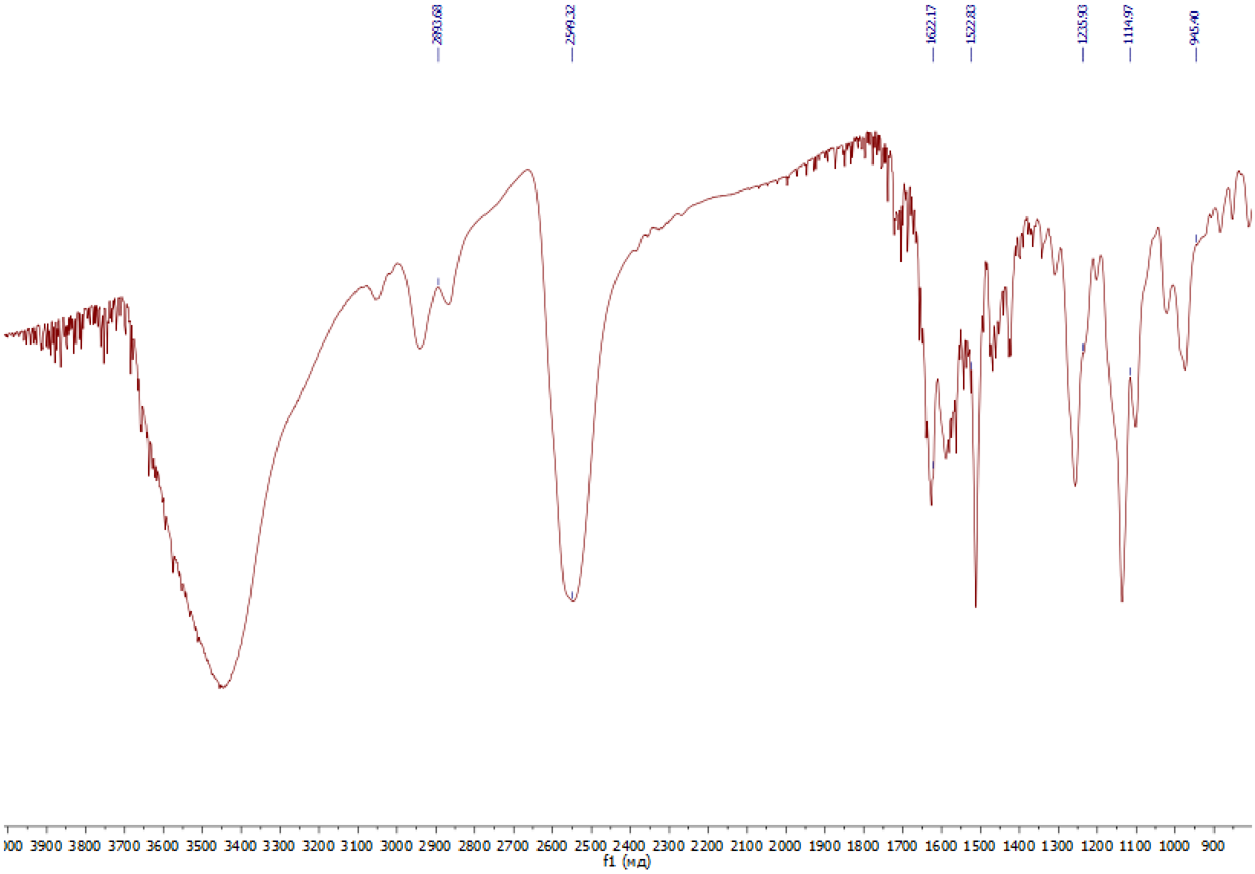

Figure S8. IR spectrum of compound **7**

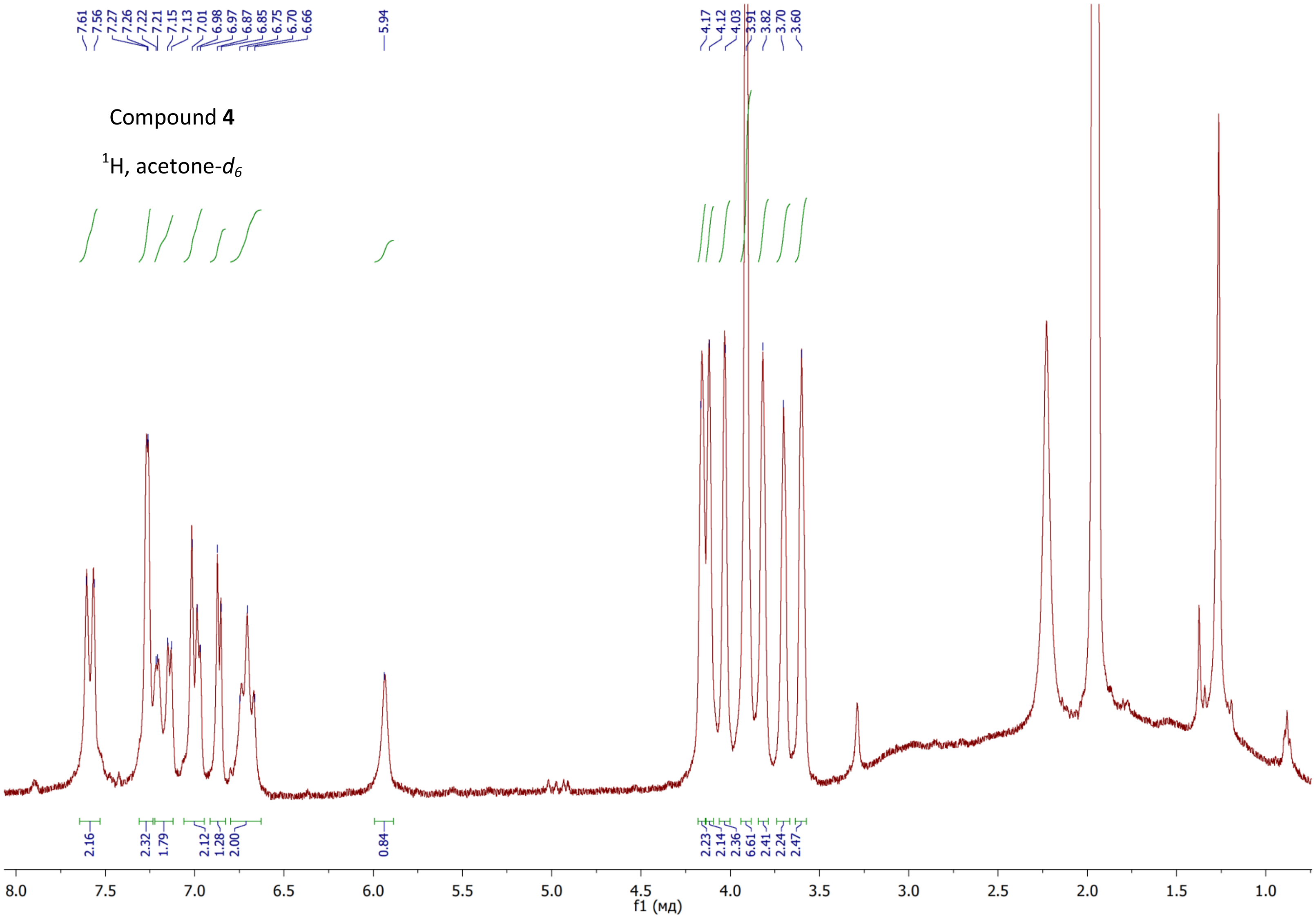

Figure S9. ^{1}H NMR spectrum of compound **4**

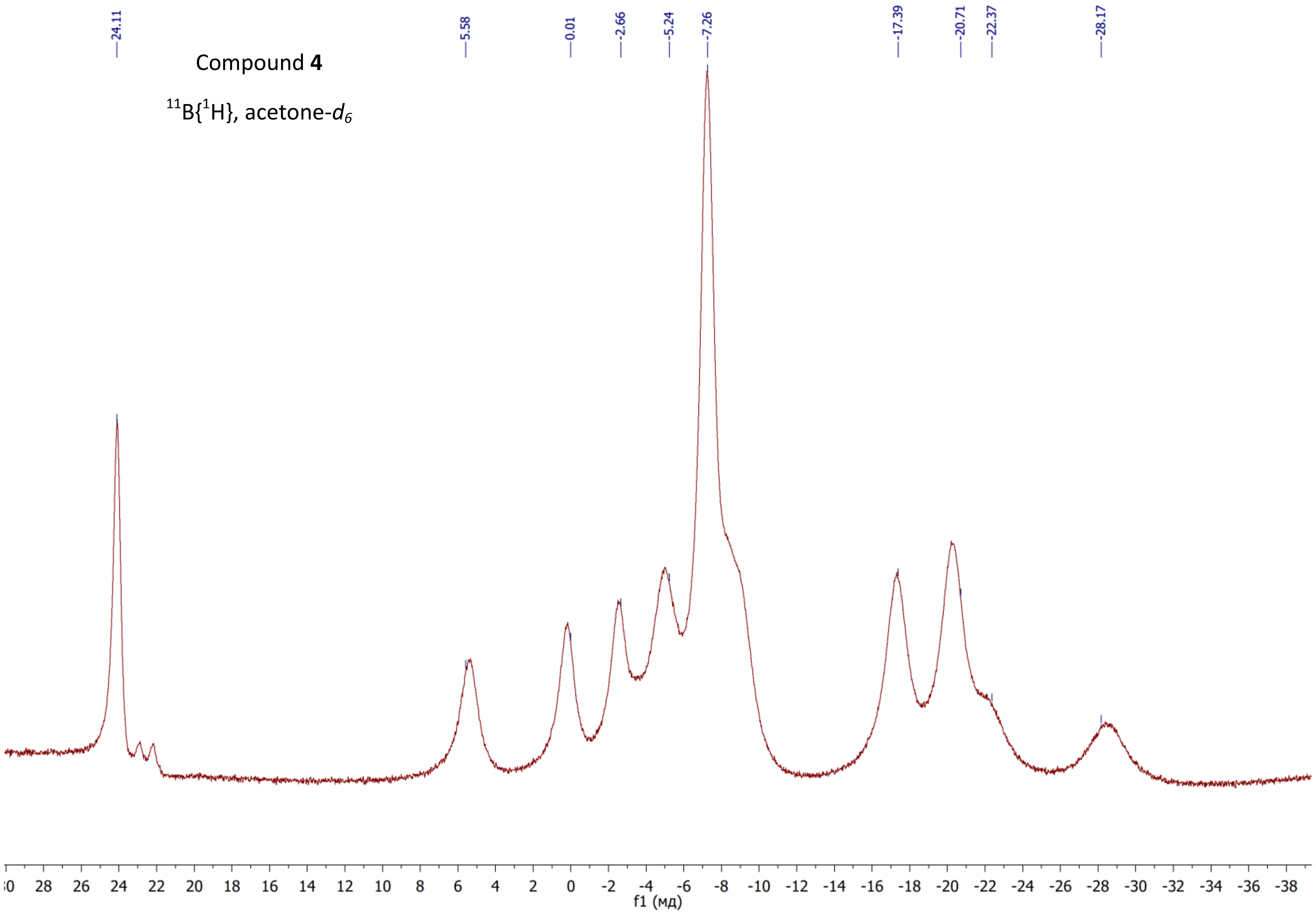

Figure S10. $^{11}B\{^{1}H\}$ NMR spectrum of compound **4**

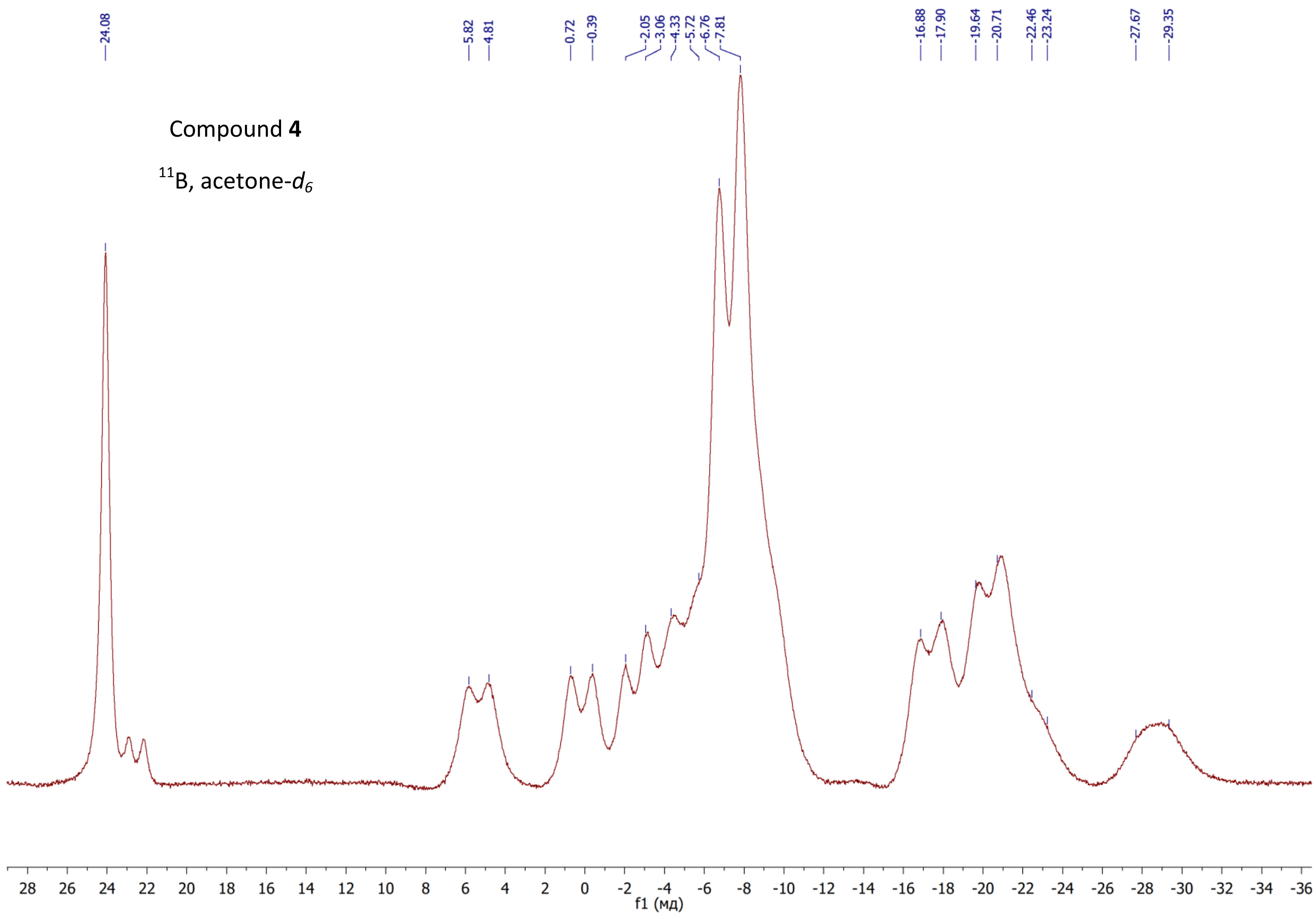

Figure S11. ^{11}B NMR spectrum of compound **4**

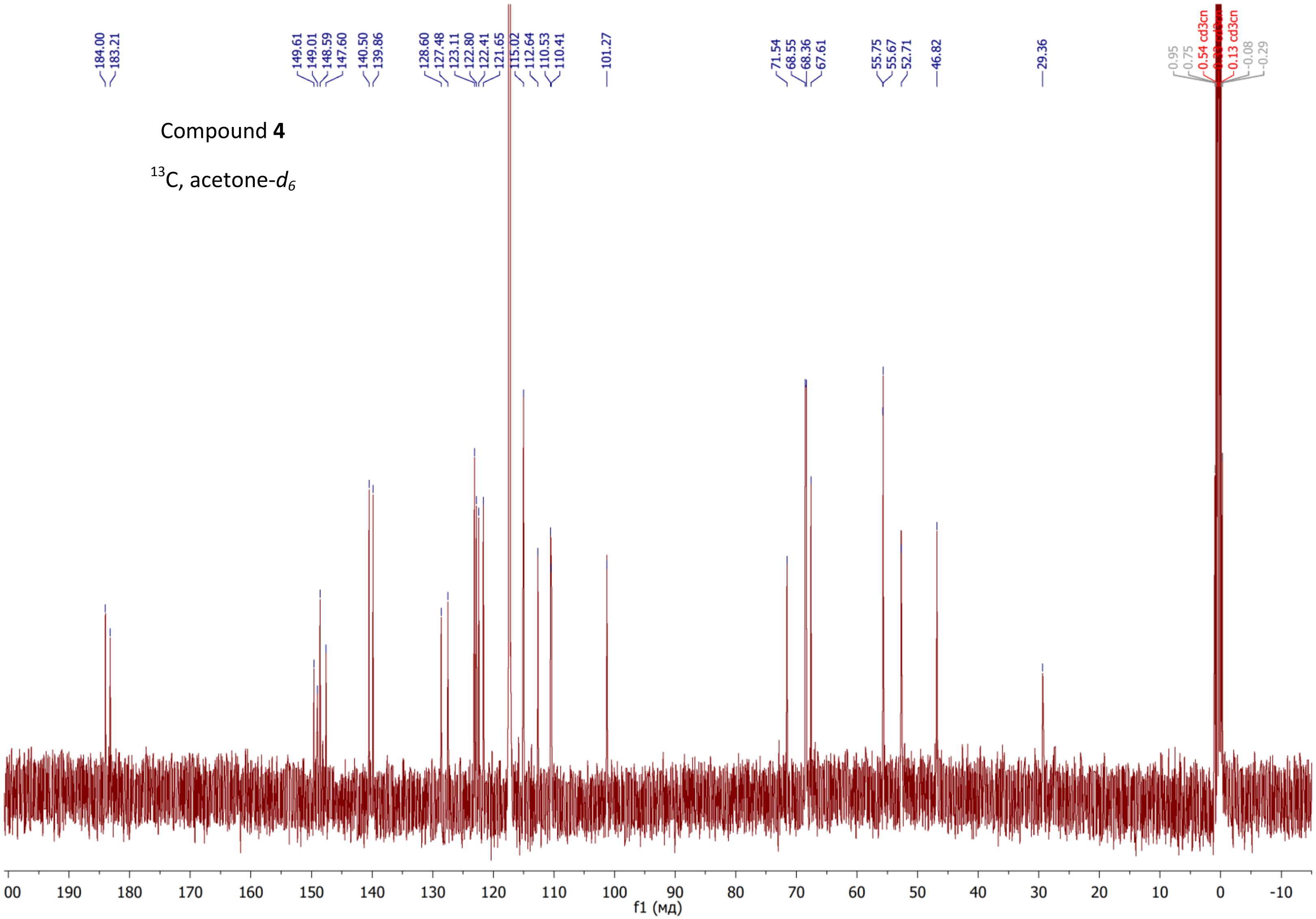

Figure S12. ^{13}C NMR spectrum of compound **4**

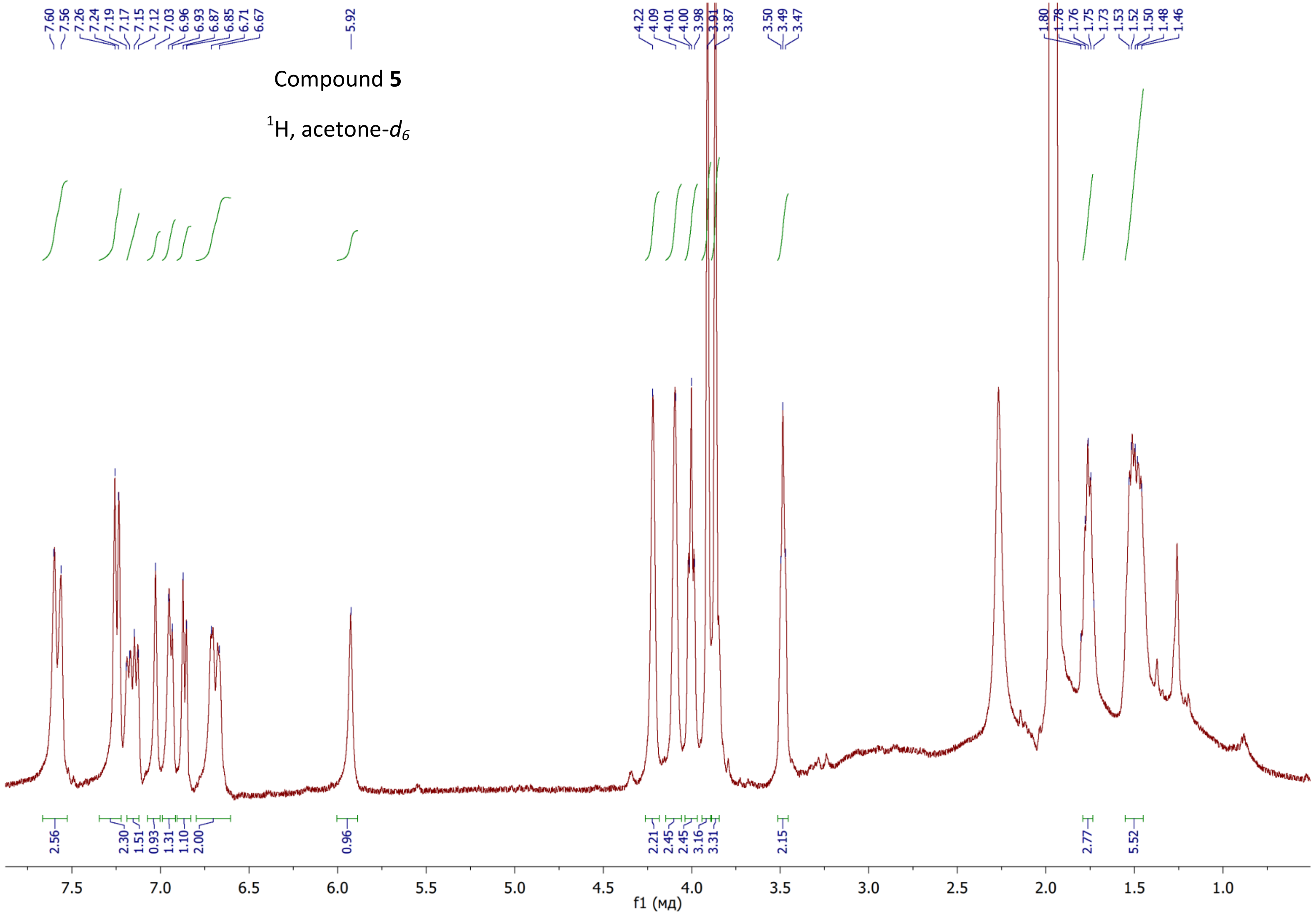

Figure S13. ^{1}H NMR spectrum of compound **5**

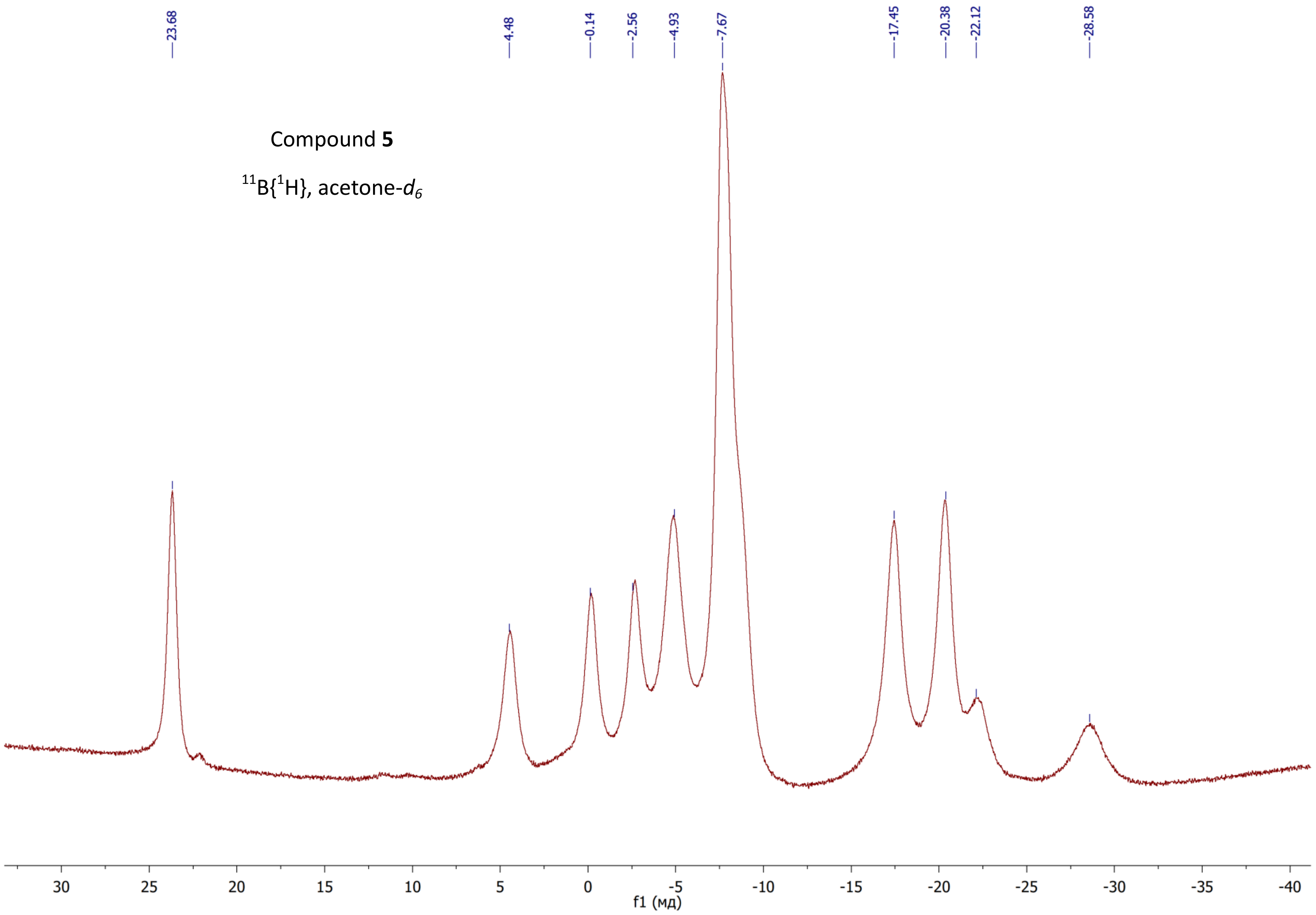

Figure S14. $^{11}B\{^1H\}$ NMR spectrum of compound **5**

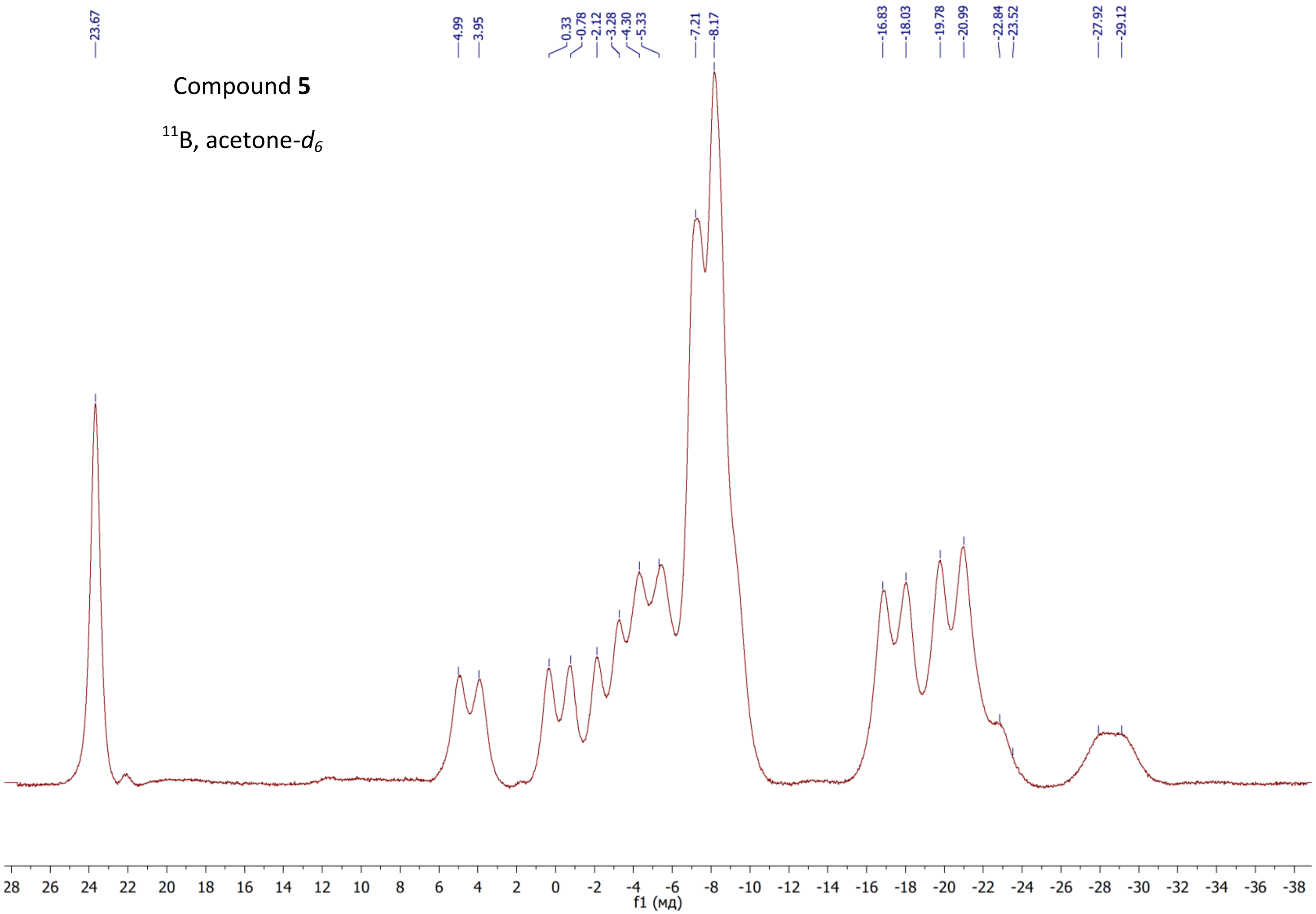

Figure S15. ^{11}B NMR spectrum of compound **5**

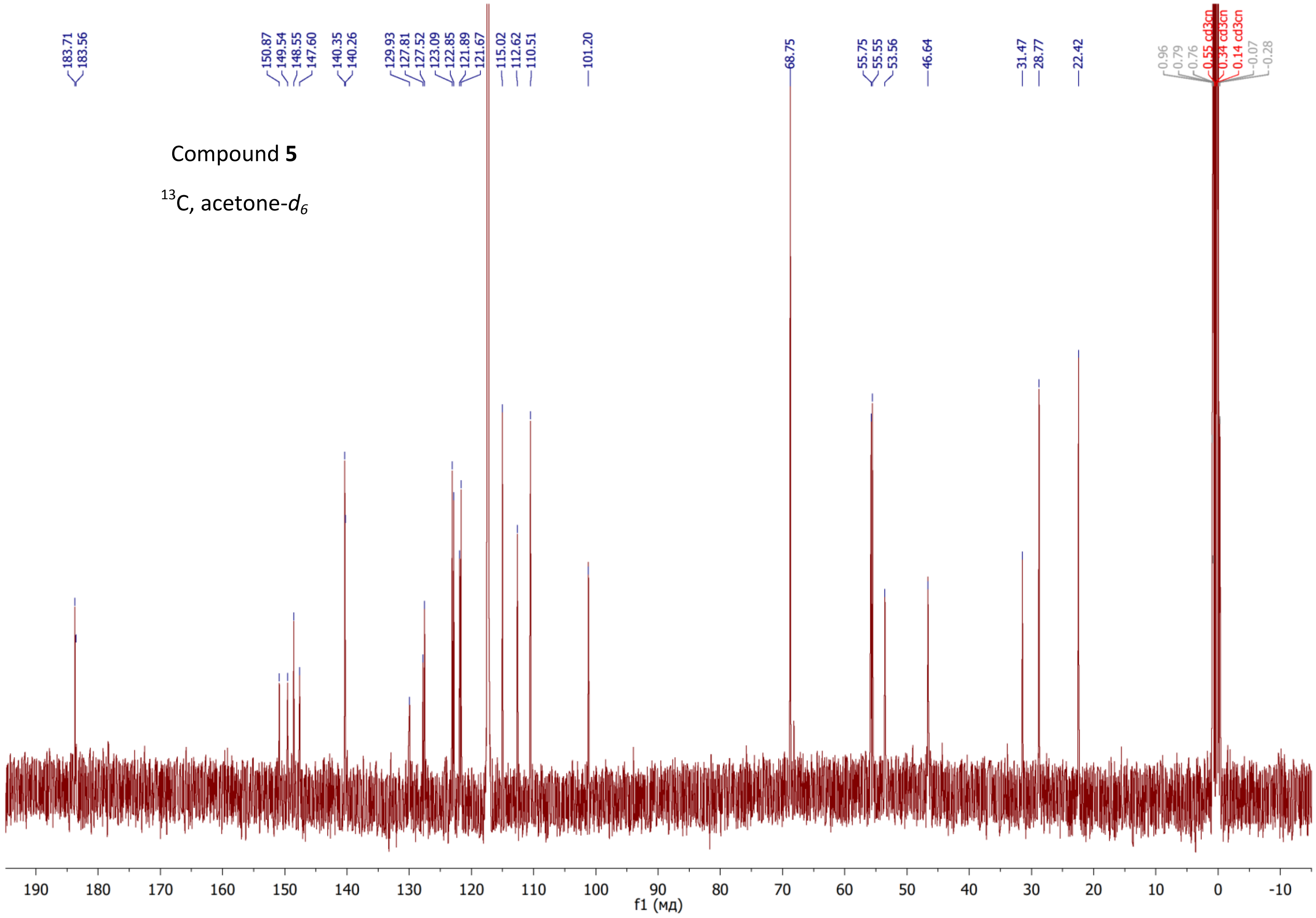

Figure S16. ^{13}C NMR spectrum of compound **5**

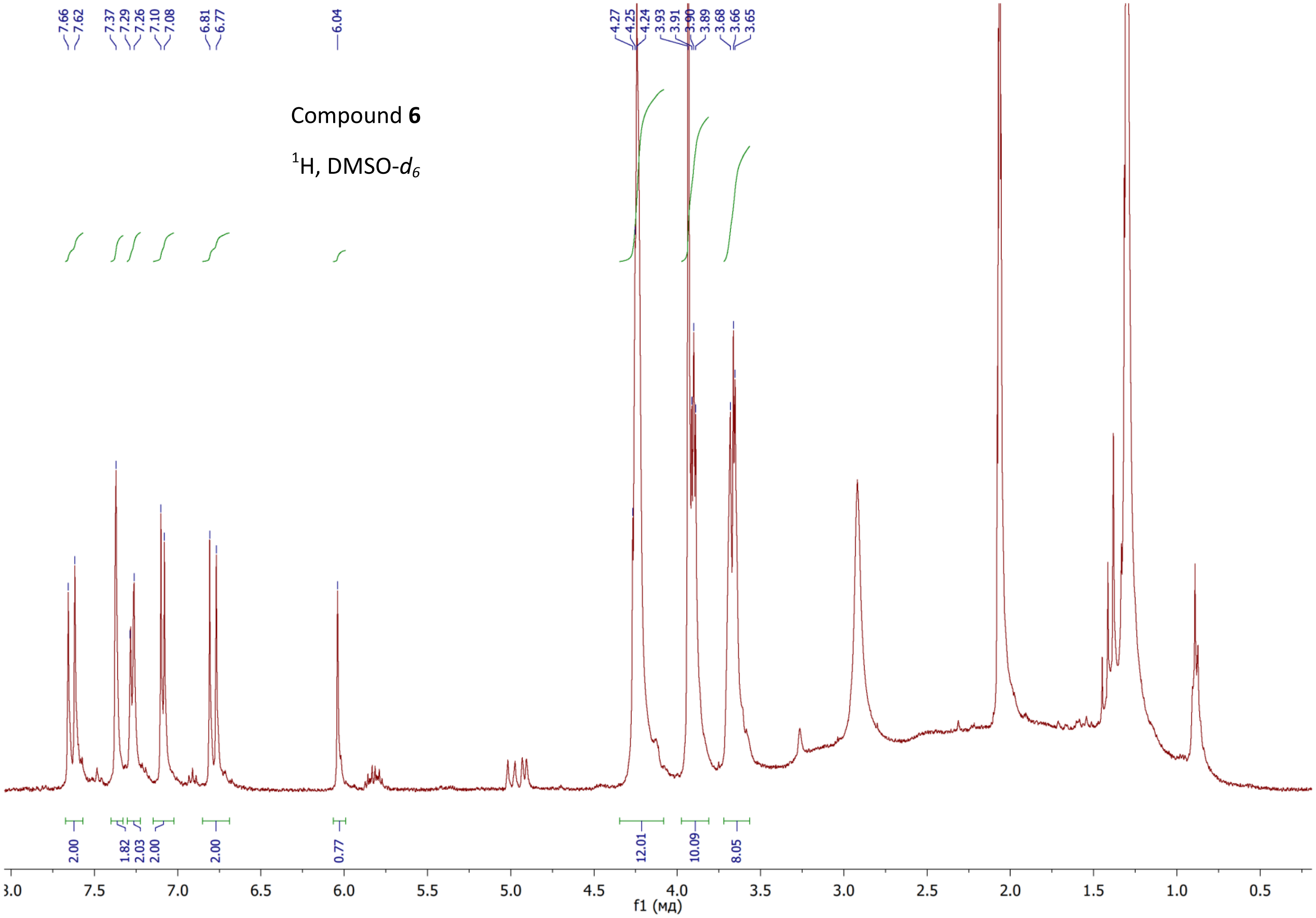

Figure S17. ^{1}H NMR spectrum of compound **6**

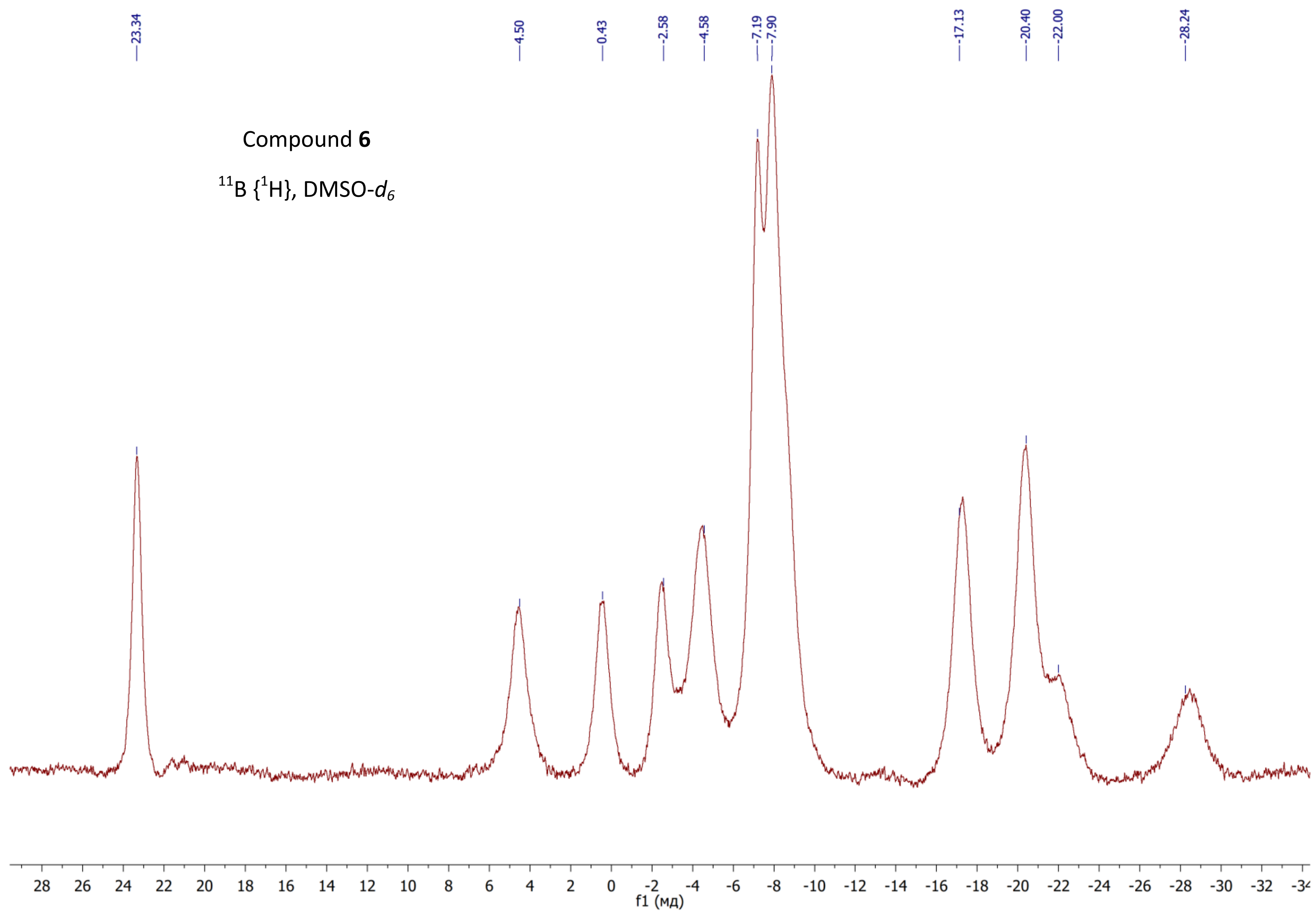

Figure S18. $^{11}B\{^{1}H\}$ NMR spectrum of compound **6**

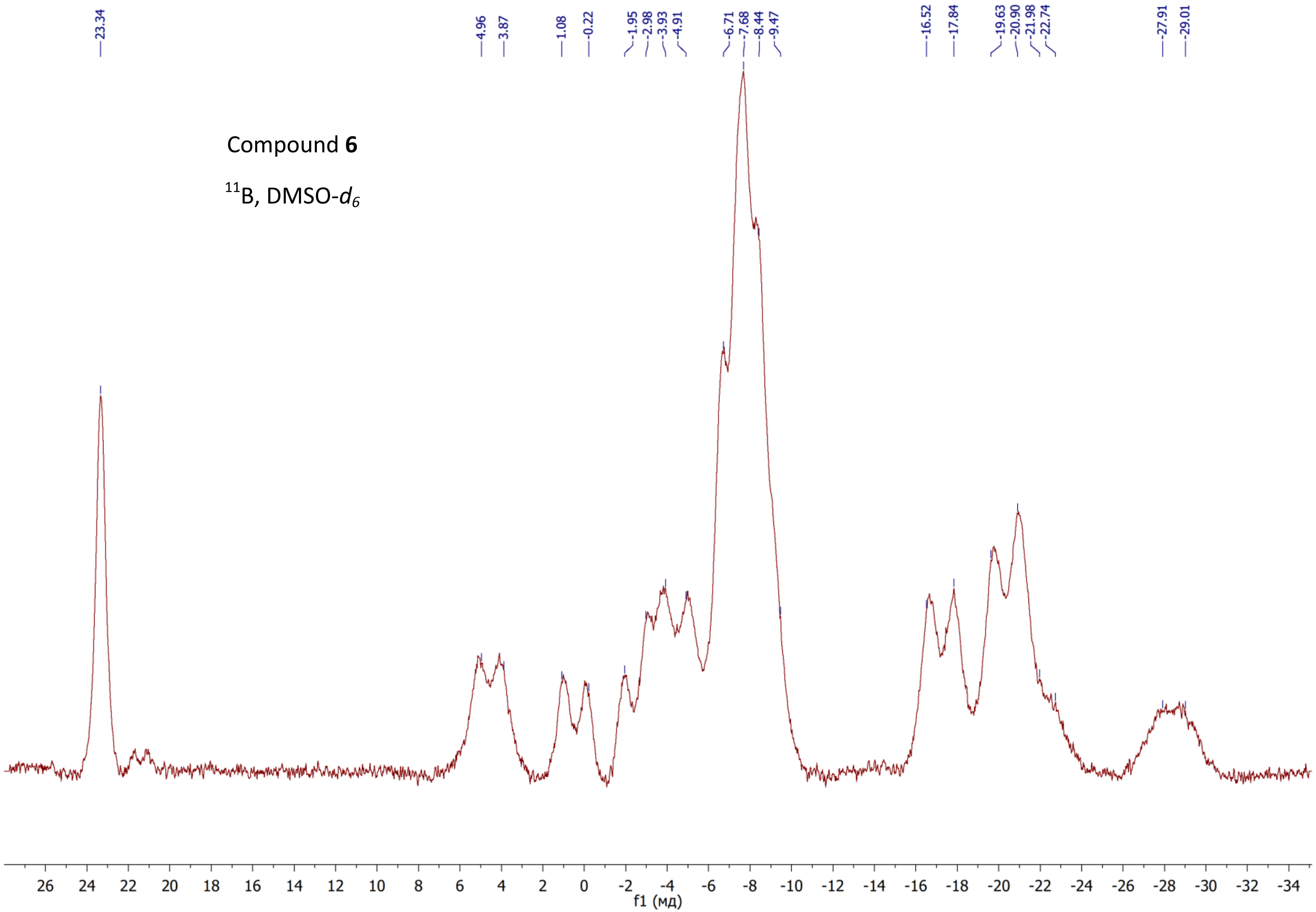

Figure S19. ^{11}B NMR spectrum of compound **6**

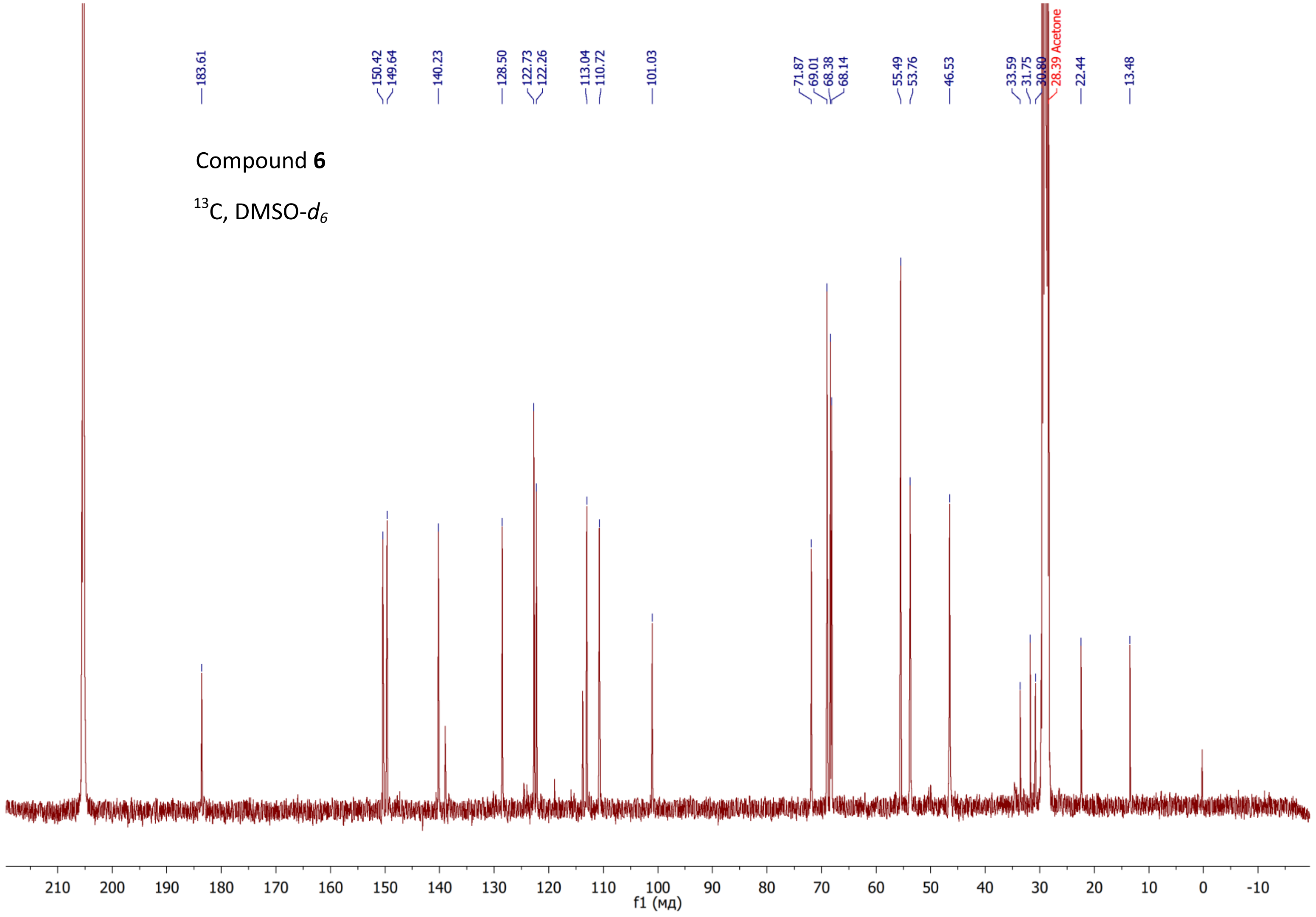

Figure S20. ^{13}C NMR spectrum of compound **6**

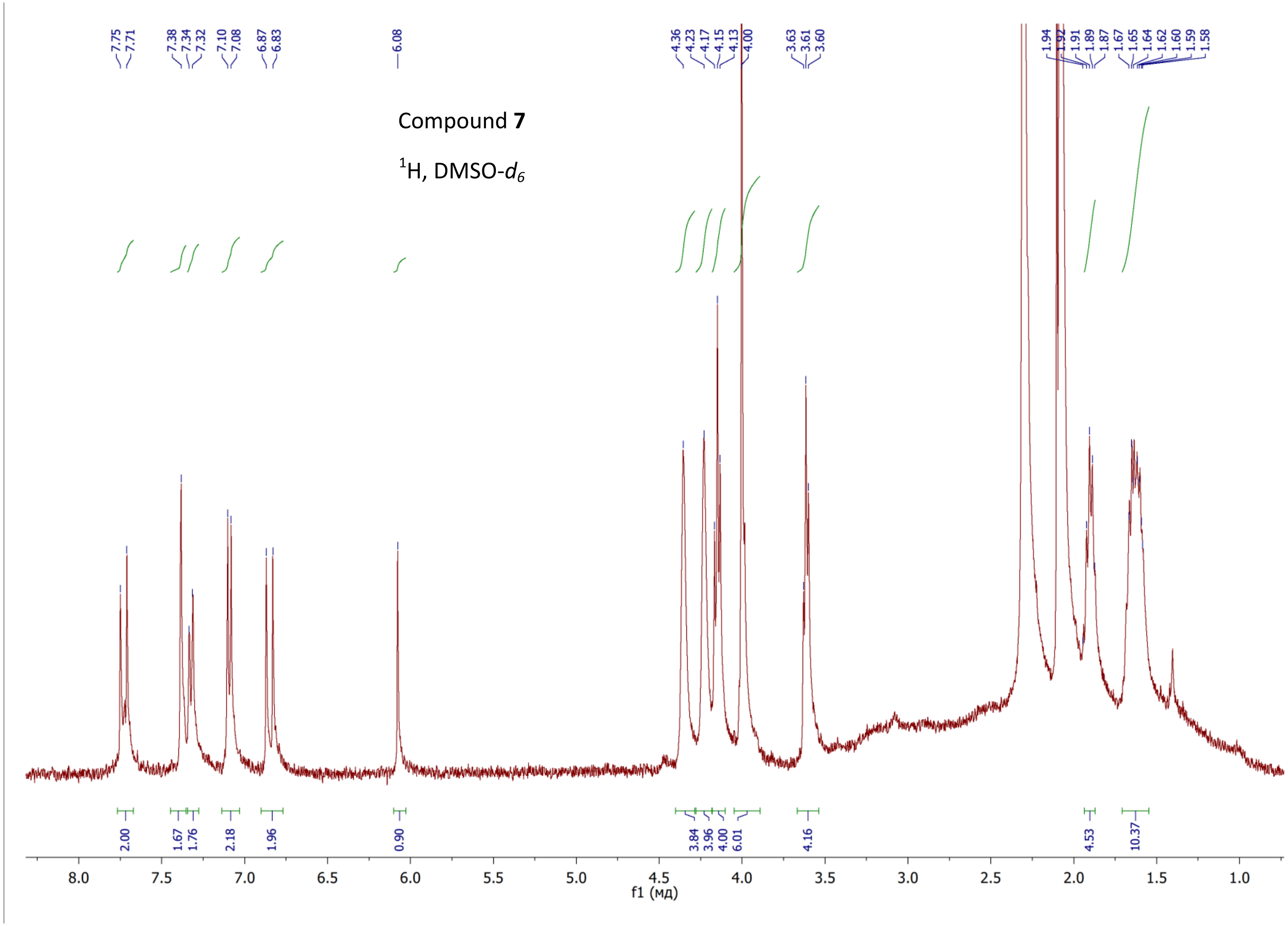

Figure S21. ^{1}H NMR spectrum of compound **7**

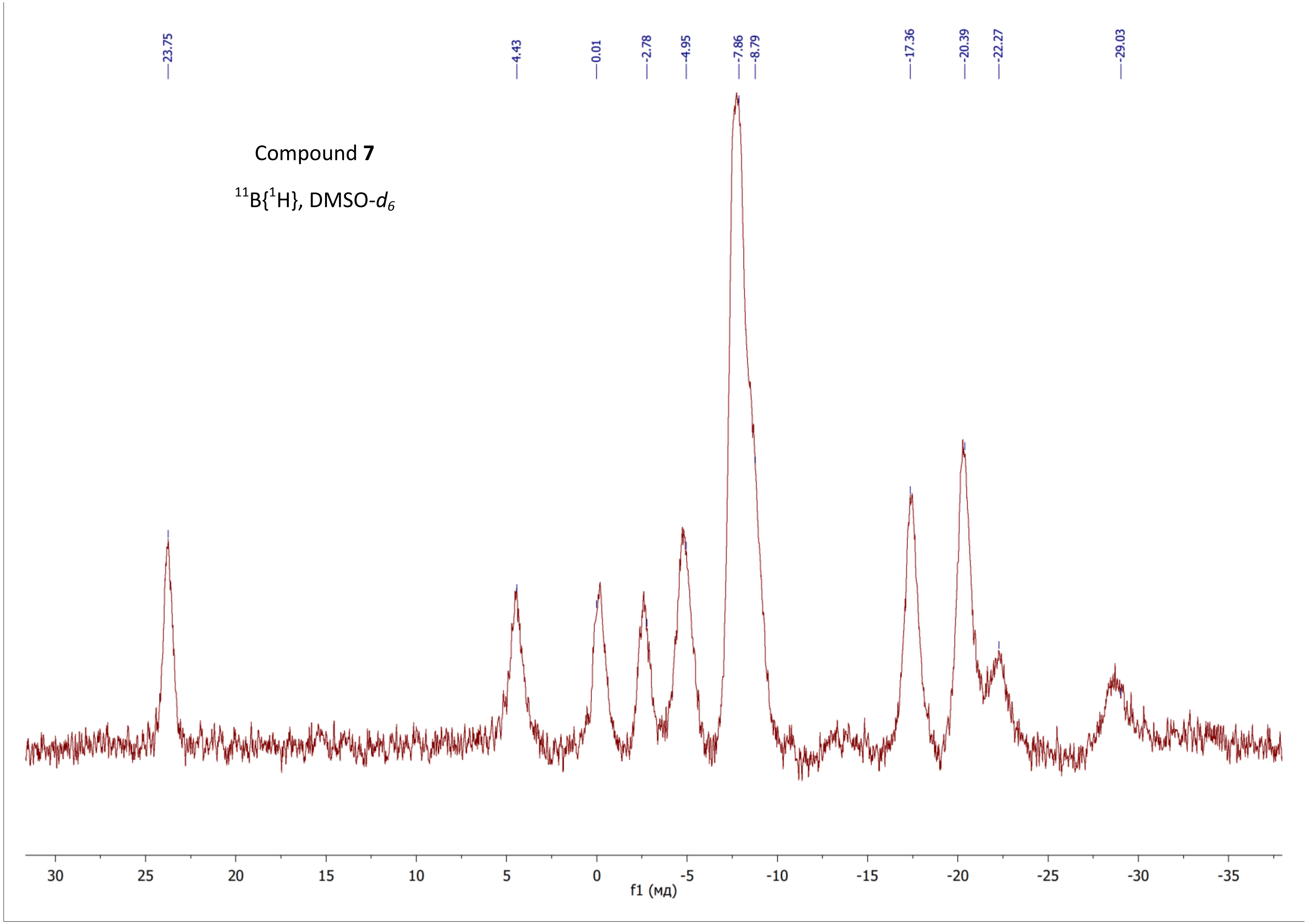

Figure S22. $^{11}B\{^{1}H\}$ NMR spectrum of compound **7**

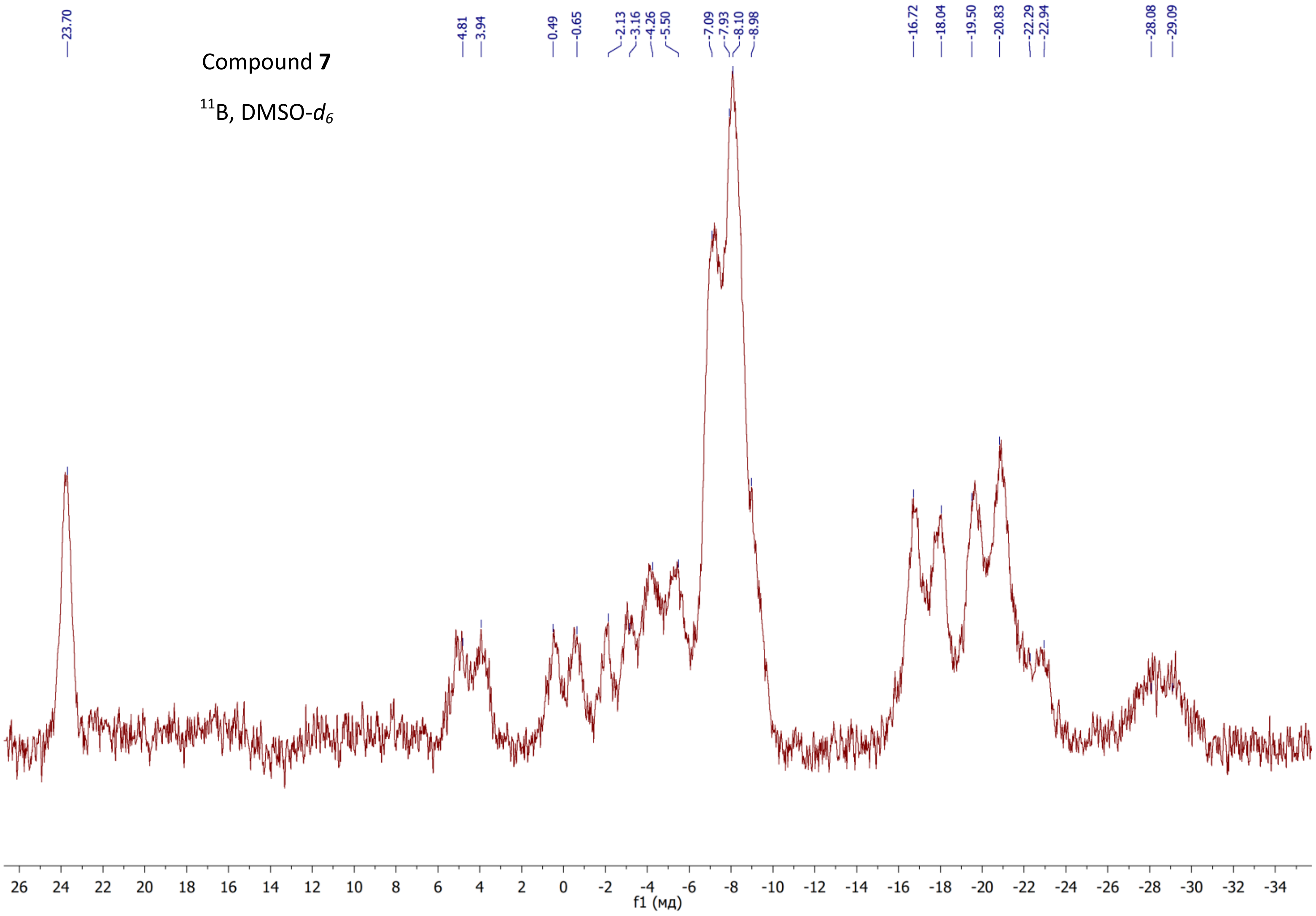

Figure S23. ^{11}B NMR spectrum of compound **7**

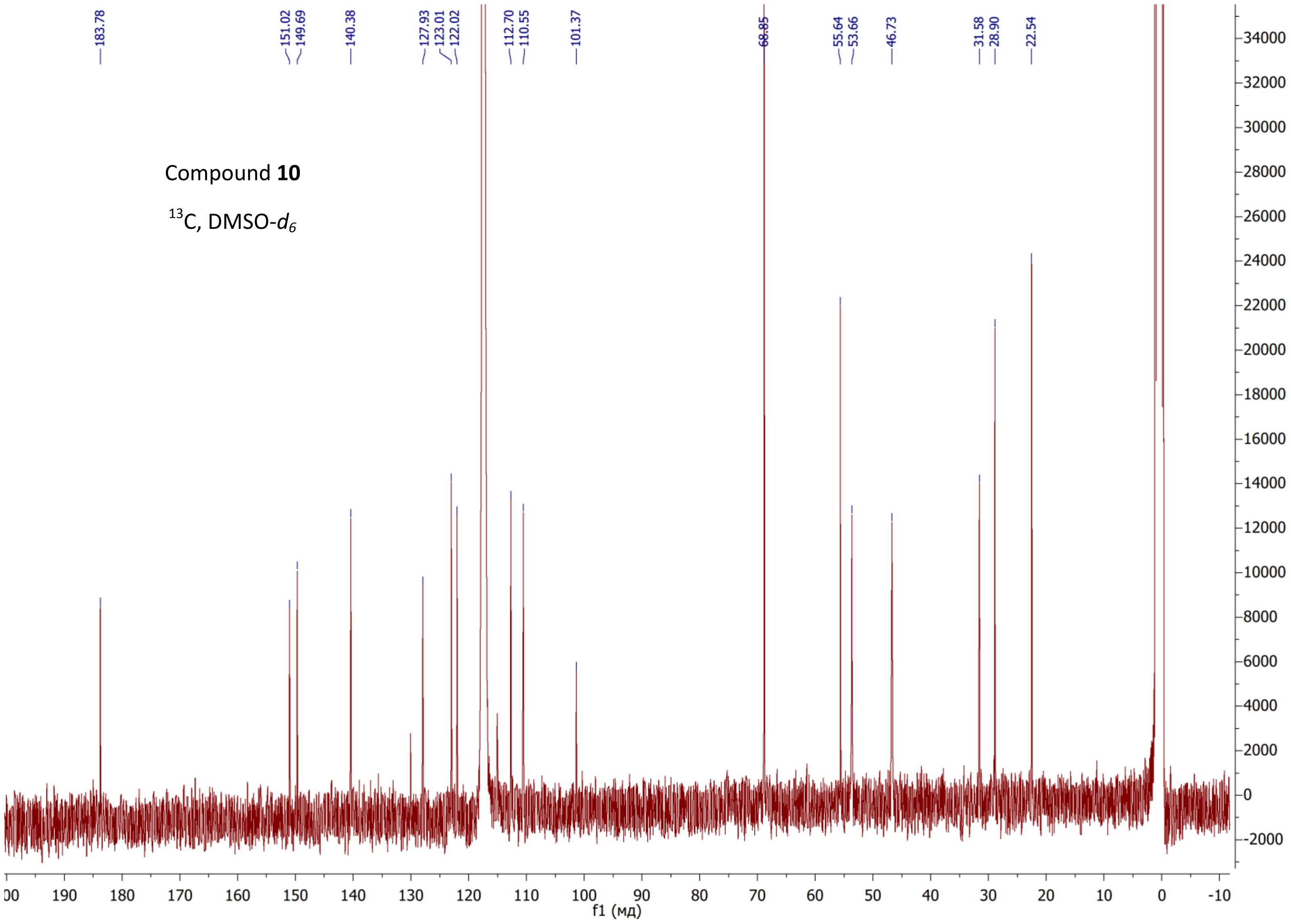

Figure S24. ^{13}C NMR spectrum of compound **7**
